# Increased sporadic extremes decrease the intraseasonal variability in the Indian summer monsoon rainfall

**DOI:** 10.1038/s41598-017-07529-6

**Published:** 2017-08-10

**Authors:** Nirupam Karmakar, Arindam Chakraborty, Ravi S. Nanjundiah

**Affiliations:** 1 Centre for Atmospheric and Oceanic Sciences, Indian Institute of Science, Bangalore, 560012 India; 2 Divecha Center for Climate Change, Indian Institute of Science, Bangalore, 560012 India; 3 Indian Institute of Tropical Meteorology, Dr Homi Bhabha Road, Pashan, Pune 411008 India; 4 Earth, Ocean and Atmospheric Science Department, The Florida State University, Tallahassee, FL 32306 USA

## Abstract

The Indian summer monsoon (ISM) shows quasi-rhythmic intraseasonal oscillations (ISO) manifested as alternate ‘active’ phases of copious rainfall and quiescent phases of ‘break’. Within these periodic phases, the daily rainfall shows large variability and exhibits spatiotemporally sporadic extreme rainfall events. The recent decades have witnessed a significant increase in the number of these extreme rainfall events, especially in the quiescent phases. This increase is accompanied by a decreasing trend in the mean monsoon rainfall and a weakening variance of its low-frequency ISO (LF-ISO) cycle. However, any physical link between this apparent paradox of increased extreme rainfall events and weakened slower-time-scale components is not yet reported. Here, using observations and numerical model simulations, we show that the occurrence of extreme rainfall events, primarily in the break phase of an LF-ISO cycle, reduce the intensity of the following active phase by stabilizing the atmosphere. We found that extreme events in a monsoon break leads to a reduction in the vertical shear of zonal winds and an increase in the static stability of the atmosphere in the following break-to-active transition and active phases. These conditions oppose the initiation and development of an active phase and lessen its intensity. This reduces the LF-ISO intensity and mean ISM rainfall.

## Introduction

It is now inarguably established that the seasonal mean and the nature of rainfall patterns in different spatial and temporal scales are changing^1–10^, and that anthropogenic attribution to these changes is beyond a reasonable doubt^11^. Recently it has been shown that the intensity of the northward-propagating LF-ISO^12, 13^ has undergone a weakening, with a significant increase in the percentage of short space-time scale extreme rainfall events, especially during the break phase over central India (CI)^5^. The changes in ISO are possibly associated with the alteration in the frequency, duration and intensity of the active and break phases^14^. However, there are still uncertainties on how the changes in short-lived extreme rainfall events translate to the changes in seasonal mean and LF-ISO. More generally, the question is whether we can associate the changes in short spatiotemporal extreme events with the changes in low-frequency variability and seasonal mean. Many researchers have attempted to understand the weakening of the monsoon, mainly in terms of large-scale circulation changes and anthropogenic activities^7, 9, 15, 16^. Few studies also documented the role of changes in land use and land cover in weakening of Indian summer monsoon rainfall^17, 18^. However, the changes in extreme rainfall events and their effects on the mean monsoon rainfall has not yet been put under the microscope. Here, we present observational evidences and model results to show how large-scale monsoonal flows and rhythmic ISO can be affected by the increase in sporadic extreme events.

Using multichannel singular spectrum analysis (MSSA)^5, 12, 19^, we filtered LF-ISO (20–60 day mode) from the daily gridded rainfall data (1° × 1°) from the India Meteorological Department (IMD) (http://www.imd.gov.in/advertisements/20160219_advt_12.pdf) for the period 1951–2013^20^. Based on the phases of the oscillatory signal, actives and breaks were identified over CI^5, 12^ (see Methods for details). Using the 99.5^th^ percentile value for May–October rainfall for the entire period at each gridpoint as the threshold, we defined extreme rainfall events over the Indian region^5^. The threshold for the extreme events at each point are chosen in such a way that the values over CI are consistent with the previous studies^1, 5^.

We attempted to mimic the scenario of increased extreme rainfall events over CI using the atmospheric component of a global climate model (GCM) and understand how it affects the seasonal mean and variability of the LF-ISO. We performed two experiments: (1) control experiment (CE) and (2) heating experiment (HE). The CE is the default simulation for ten years. In the HE, we prescribed heating in the atmosphere over CI in such a way that it simulates the conditions for increased extreme rainfall events, with dominant relative increase in the break phase. The heating profile and its space-time distribution were obtained from the analysis of extreme rainfall events over CI using observational/reanalysis data. Such impulsive short space-time scale atmospheric heating in the model destabilizes the atmosphere within a very short period and makes it conducive for convective processes (see Methods for model details and supplementary information (SI) for experimental design). We started the model HE every June 1^*st*^, using the restart files from the CE. Each simulation, then, continues for 5 months and ends on October 31^*st*^. These short simulations were necessary because after the heating was imposed in the model, the HE deviated from the CE and if we integrated long enough, the oscillatory signals might go entirely out-of-phase in two simulations. The experiment is carried out for ten years to rule out the possibility that the changes observed could be outcome of internal variability. Here, we present the results obtained from observational analysis and numerical simulations on how change in the number of extreme rainfall events is associated with the changes in LF-ISO and the seasonal mean rainfall.

## Results

**Figure 1:**
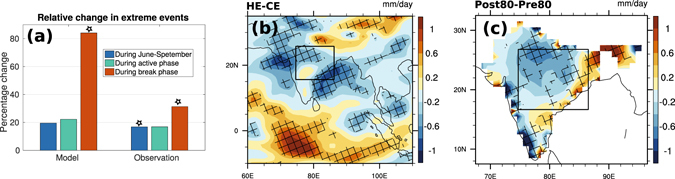
Changes in extreme events and daily climatology during JJAS. (**a**) Relative changes in the occurrences of extreme events over CI in model and observations during monsoon season, and active and break phases. Change in daily climatology in (**b**) Model (HE–CE) and (**c**) Observations (post80–pre80), during JJAS months. Units are in *mm*/*day*. Asterisks in (**a**) and hatched regions in (**b**) and (**c**) indicate places where the changes are significant at 10% level (using t-test). Rectangles in (**b**) and (**c**) mark the central Indian (CI) region (16.5°N to 26.5°N, 74.5°E to 86.5°E). Figure (**a**) is generated using MATLAB R2015a (https://in.mathworks.com/products/new_products/release2015a.html). Figures (**b**) and (**c**) are generated using NCAR Command Language 6.3.0 (http://www.ncl.ucar.edu/).

Observational analysis suggests that the number of extreme rainfall events over CI has significantly increased in the post-1980 (1981–2010; post80) period compared to the pre-1980 (1951–1980; pre80) era during the months from June–September (JJAS), with the dominant change observed in the break phase (Fig. 1a)^5^. The JJAS seasonal mean rainfall also shows a significant decrease in recent times over the CI region (Fig. 1c).

In the model, we attempted to simulate similar scenario of increased extreme events during the break phase. To do so, heating was prescribed primarily during the break phase of the LF-ISO cycle, as per the data obtained from the CE (Fig. 1a). In fact, to focus on the importance extreme events during break phase, we increased the number of extreme events in the HE breaks higher than that in observations. Consistent with the observational studies, the JJAS seasonal mean rainfall over most of the Indian land region including CI and also over the Bay of Bengal (BoB) and the northern Arabian Sea (AS) has significantly reduced in the HE as compared to the CE (Fig. 1b). This, with the increase in rainfall over the equatorial Indian Ocean (EIO) indicates the weakening of the Hadley cell circulation over the Indian region, which has been documented in a few earlier studies^7, 21, 22^. Along with an increase in extreme rain events, we observe a decrease in moderate rain events over the CI region, which matches with the observational studies^1^ (SI).

**Figure 2:**
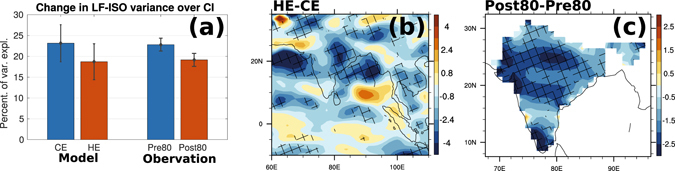
Change in strength of low-frequency ISO mode. (**a**) Explained variance of LF-ISO over central India from model (CE and HE) and observations (pre80 and post80). Both for model and observations, changes in mean are significant at 5% level (using t-test). Change in spatial distribution in the percentage of the variance explained (to the total rainfall) by LF-ISO from (**b**) Model and (**c**) Observations, during JJAS months. Hatched regions indicate regions where the changes are significant at 10% level (using t-test). Figure (**a**) is generated using MATLAB R2015a (https://in.mathworks.com/products/new_products/release2015a.html). Figures (**b**) and (**c**) are generated using NCAR Command Language 6.3.0 (http://www.ncl.ucar.edu/).

Naturally, the question arises as to how the changes in short space-time scale extreme events in the break phase over CI alter the seasonal mean rainfall? A simplistic approach to answer this question would be to look into how the increased extreme events affect the active-break cycle. Consistent with the weakening of the LF-ISO variability documented in an earlier study^5^, the model experiments show a marked difference in the post80 as compared to pre80 values (Fig. 2a). The reduction in LF-ISO variance is not only confined over CI but is spread across India (Fig. 2b and c). The reduction is also observed over the northern BoB and AS where the seasonal mean rainfall has reduced in the model. The changes in the equatorial region are not significant, even though a weak increase is observed over the central equatorial Indian Ocean (CEIO). This indicates that the intensity in the 20–60 day scale has lessened, which can be a manifestation of either disrupted break phases (increased extreme events) or less intense active spells. However it is paradoxical how increased rainfall during breaks, in terms of extreme events, cause decrease seasonal mean rainfall. We hypothesise that the increase in irregular extreme rainfall events, with highest relative increase in breaks, modulates the rainfall in the active phases. This, in turn, engenders the reduction in seasonal mean rainfall over CI.

The hypothesis we made that increased extreme rainfall events in breaks can attenuate the rainfall in the active phase is justified by the fact that in both observations and model experiments, the total rainfall in the active phase over CI has reduced significantly in recent times (SI). The most pronounced changes are seen over the adjacent oceanic regions compared to land in the model. Furthermore equatorial rainfall during the active phase as well the break phase has increased, which is in agreement with the increase in seasonal mean rainfall over the EIO. We also noted that the change in the seasonal mean as well as the active phase rainfall shows a dipole structure over India: increase over the north-eastern region and decrease over CI. This is again consistent with the model results and matches with previous observational studies^10^.

**Figure 3:**
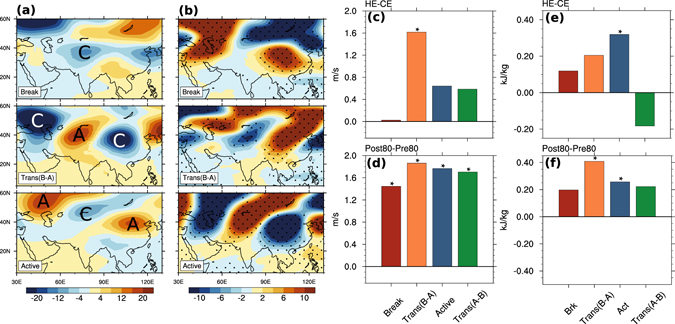
Possible mechanism for change in rainfall. Anomalous geopotential height at 200hPa during (**a**) break, transition (break-active), and active phases in the CE. (**b**) Same as (**a**) but for HE–CE, showing the change in HE compared to CE. Change in vertical wind shear (*U*_200*hPa*_ − *U*_850*hPa*_) averaged over the northern Indian region (20°N to 35°N and 65°E to 95°E) in different phases (break, transition (break-to-active), active, and transition (active-to-break), respectively) from (**c**) Model and (**d**) Observations. Change in VMS over the CI region in different phases from (**e**) Model and (**f**) Observations. Observational analysis here is done using NCEP-NCAR Reanalysis-1^35^ data. Trend in global mean temperature is subtracted before calculating VMS. Units are in *m* for geopotential height, *m*/*s* for wind shear, and *kJ*/*kg* for VMS. Dotted regions in (**b**) and asterisks in (**c**–**f**) indicate where the changes are significant at 10% level (using t-test). ‘A’ and ‘C’ in (**a**) and (**b**) indicate the anticyclonic and cyclonic structures of winds at upper troposphere, respectively. Figures are generated using NCAR Command Language 6.3.0 (http://www.ncl.ucar.edu/).

It is now important to understand the factors that might have played a role in the reduction of rainfall in active phase, and contributed to the decreased intensity of LF-ISO and seasonal mean over CI. In other words, we need to understand what triggers large-scale convection over CI and how it has changed. We noticed that in the break–active transition phase (trans(B–A)), a strong positive anomalous geopotential high is formed over the central Asian region (Fig. 3a). This anomalous high is associated with a strong easterly vertical shear over the Indian region^23^. Some studies have suggested that the easterly vertical shear destabilizes the Rossby waves over the equatorial region^23, 24^. The presence of boundary layer friction provides available potential energy, which further increases the moist Rossby wave instability^25^. These effects generate convective activity over the region^23, 25^. Typically, the establishment of anomalous high leads to the generation of convection within 4–5 days (as it is the typical length of a break–active transition before the establishment of an active phase over CI). The area-averaged vertical shear of zonal winds over northern India shows significant weakening during all the phases in observation (Fig. 3d), with the maximum change seen in the trans (B–A) phase. Similarly, a significant change in the vertical shear was also observed in the model (Fig. 3c). Significant changes in the magnitude of anomalous geopotential high was also observed (Fig. 3b), possibly implying a shift in the phase of the Rossby wave. This reduction in shear plays a crucial role in the reduction in the amount of rainfall in the following active phase as the Rossby wave instability is weaker in the HE, as discussed earlier. Hence, we conclude that weakening of the easterly vertical shear in the trans(B–A) phase affects the following active phase by lessening the favourable conditions for the growth of instability and generation of convection over the CI region.

Large-scale convection is associated with stability of the atmosphere, which can be measured by vertical moist stability (VMS). VMS is defined as the difference between the column integrated moist static energy (MSE) of the upper and the lower troposphere. It provides a necessary condition for the formation of deep convective clouds^26, 27^. We found that during the active phase, the VMS over CI has significantly increased in the HE as compared to the CE, making the atmosphere more stable. This is consistent with the substantial decrease in the active phase rainfall. The increase in VMS is also observed in the break and trans(B–A) phases. In agreement with the decrease in rainfall, the JJAS mean VMS shows an increase over CI, the northern BoB and AS (SI). On examining the dry static energy and comparing it with MSE, we found that moisture plays the dominant role in this change in stability (SI). We note that the magnitude of changes seen in different phases are slightly different in model and observation. This is possibly because of (1) using reanalysis datasets for composites based on ISO phases defined by the IMD rainfall data and (2) influence of many other factors, including rising sea surface temperature (SST) in observations. However, the model captures the changes in the large-scale spatial patterns quite well (not shown).

## Conclusions and Discussion

**Figure 4:**
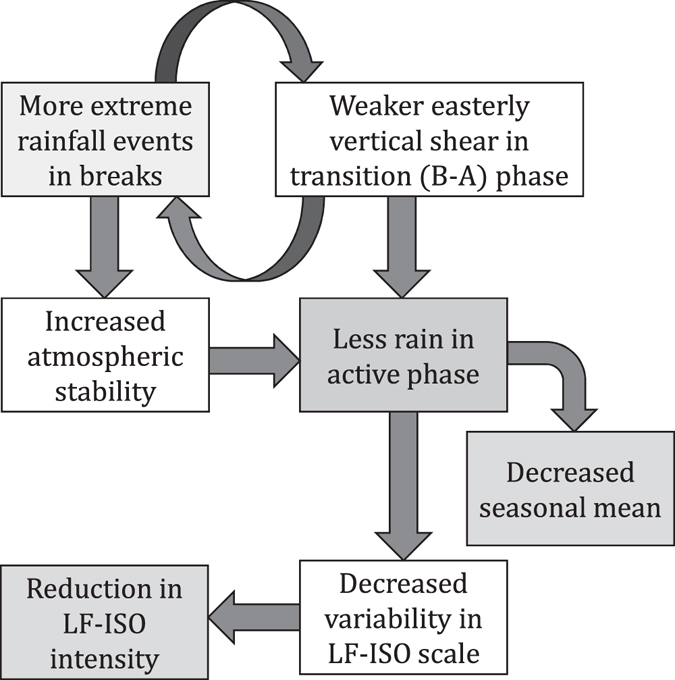
Mechanism in a flowchart. Flowchart for the mechanism of the decrease in seasonal mean and low-frequency intraseasonal variability strength over India. Increased extreme rainfall events in the break phases weakens the easterly vertical shear over the northern Indian region. This weakens the instability over the region, causing the lesser convective activity in subsequent active phase. This fact is supported by the the fact that the stability over CI has increased when we have more extreme events, especially during breaks. Weaker vertical shear creates favourable conditions for the growth of deep convective storms, providing a feedback in process. Since majority of the seasonal mean rainfall occurs during the active phase, this decrease in active phase rainfall decreases the seasonal mean. Reduction in active phase rainfall also incapacitates variability in low-frequency intraseasonal scale, which in turn, weakens the LF-ISO intensity. Figure is generated using Microsoft Powerpoint 2010 (https://products.office.com/en-us/microsoft-powerpoint-2010).

Using observational analysis and numerical modeling simulations, our study presents evidences that sporadic extreme rainfall events affect the low-frequency intraseasonal quasi-rhythmic nature of rainfall and seasonal mean during the monsoon over India by changing the vertical shear of zonal winds and the stability of the atmosphere (Fig. 4). Increased extreme events not only decreased the daily mean rainfall during the active phase, but also reduced the total number of active days in the HE (SI). This with the increase in total break days indicate that the LF-ISO rhythm has changed in both magnitude and phase. A similar analysis was also performed with the high-frequency intraseasonal mode (10–20 day), but we did not find any change in the intensity of this mode in recent decades (SI), which is consistent with a previous study^5^.

The decrease in vertical shear can also provide favourable conditions for the growth of deep convective storms over the region, thus providing a feedback in the increase of extreme rainfall events. However, the linkage between deep convective storms and extreme rainfall events is still a mystery^28^, and the response of extreme rainfall events to the change in vertical shear needs to be understood clearly. We also found that monsoonal rainfall over the Sahel region and east Asia have increased in the HE compared to the CE (SI). The increase in rainfall in recent decades over these regions has been documented in several observational studies and Coupled Model Intercomparison Project (CMIP5) future projections^29, 30^, but there is no consensus on the causes.

We also note that reanalysis datasets shows significant weakening of easterly vertical shear in all the phases of LF-ISO. This reflects the fact of weakening of the June–September mean easterly vertical shear^5^. This can be an outcome of weakening of the tropical easterly jet (TEJ)^31^. Which is again attributed to the decreasing trend in the upper tropospheric meridional temperature gradient over the region. Still, the maximum change in the shear is observed in the trans(B–A), which might be instrumental in trigering convection. However, we have not incorporated these changes (and any other that influences the TEJ) in the model. More specific modeling studies might be required to address these issues in the context of extreme rain events and ISOs.

It has been documented in few studies that the seasonal mean rainfall may increase in future under the impact of enhanced anthropogenic greenhouse gas (GHG) emissions^9, 32^. Enhanced moisture may play a role in this increase, however, weakened large-scale circulation over the south Asian region can neutralize this effect in some extent. Also, the aerosols can have a significant impact on the monsoon rainfall over India^33^. Regional aerosol heating can have large impact on the phase of upper tropospheric Rossby wave in pre-monsoon season^27^. It was also shown that the rainfall intensity can change the aerosol concentration over India, which can lead to the changes in cloud properties^34^. Therefore, the aerosols can modulate the LF-ISO cycle over India. In this paper, we neither have incorporated changes in greenhouse gas emissions nor aerosol forcings. Our experimental design is completely focused on understanding the changes in mean and intraseasonal variability in monsoon rainfall, to an increase in the number of extreme events. Therefore, the effects of increased GHG or altered aerosol forcings are not present in our simulations.

Since our intention is not to represent the actual scenario of the changing nature of monsoon while prescribing atmospheric heating, present-day SST was incorporated in the model in order to isolate the direct effects of extreme rainfall events on the monsoonal flow. In fact, we used monthly varying climatological SST in our simulations to remove interannual effects from SST. We note that ISO intensity shows significant correlation with SST over various regions in an interannual timescale^13^. Coupled model simulations can be done at a later stage to understand how the processes like air-sea interaction may change if extreme rainfall events are increased. We do not provide answers as to why extreme rainfall events have increased over India. Rather, our modeling experiments establish how the seasonal mean and intraseasonal behaviour would change if the number of extreme rainfall events increase. The attribution of extreme rainfall events to climatic/anthropogenic factors is a different problem to be dealt with and requires meticulous modeling studies. Numerical models for monsoon prediction must simulate extreme events realistically in order to improve their skill in all time-scales.

## Methods

### Observational data

We used the IMD gridded rainfall data from 1951–2013 for the observational studies on rainfall variability^20^. We also used the National Centers for Environmental Prediction–National Center for Atmospheric Research (NCEP–NCAR) Reanalysis-1^35^ zonal wind, temperature, specific humidity and geopotential height datasets for calculation of vertical shear and VMS over the Indian region (https://www.esrl.noaa.gov/psd/data/gridded/data.ncep.reanalysis.html).

### Extraction of intraseasonal modes from rainfall data

The daily rainfall data (May–October) from model and observation were used for extraction of intraseasonal modes. The daily climatology was removed from the respective datasets to obtain the anomalies. A 5-day moving mean was applied to eliminate very high-frequency variability. Then the 184-day long data (for each year) was feed into the MSSA algorithm, with a window-length of 60 days^19^. MSSA is a very useful data-adaptive technique to analyze the spatiotemporal behaviour of short and noisy timeseries and has been used quite frequently in climatic applications and monsoon variability studies^5, 12, 36^. Using MSSA, we reconstructed low- (20–60 day) and high-frequency (10–20 day) modes from the rainfall anomaly data. Based on the phase angle of the oscillatory modes we defined the active, break and transition phases of low-frequency ISV over CI. We note that, longer data (May–October; 184-days) were required for the ISO modes extraction using MSSA. However, subsequent analysis are all done using June–September data. We refer to an earlier work for the detailed methodology for the extraction of intraseasonal modes and active-break cycle^12^. Although the number of active and break days are higher than that identified in previous studies)^37^, the phases we found are in quite good agreement with theirs (see SI).

### Choice of model and configuration

We used the Community Earth System Model version 1.2 (CESM1.2)^38^, developed at the NCAR (http://www.cesm.ucar.edu/models/cesm1.2/) in our study. This is the latest version of the model at present and is widely used in understanding the climate over different regions of the world. CESM is basically a coupled climate model composed of several components that simultaneously simulate the Earth’s atmosphere, ocean, land, land-ice and sea-ice; it also has a central coupler component. In our study, we do not involve the active ocean component since our goal was to understand the association of ISV with extreme rainfall events over the Indian region. We prescribed monthly-varying climatological SST (data ocean (DOCN)) and sea-ice (1982–2001)^39^, while keeping the atmospheric and land components active. The atmospheric and land components of this model are Community Atmosphere Model version 5 (CAM5) and Community Land Model version 4.0 (CLM4), respectively. We set the initial conditions as the values in the present day; values of aerosols, carbon dioxide and other greenhouse gases were set to the present day levels. In summary, we used active atmosphere (CAM5), land (CLM4), and river transport model (RTM) with stub glacier and prescribed data ocean (DOCN) and sea-ice. The atmosphere, land, sea-ice and data ocean components communicated through the coupler every 30 minutes. We used a horizontal resolution of finite volume 0.9° × 1.25° in atmosphere component (the land model also has the similar horizontal grid) for our analysis. The vertical coordinate of the atmosphere is a hybrid sigma-pressure system, where the upper regions of the atmosphere are discretized by pressure only and the lower levels are given by sigma vertical coordinate. There are 30 vertical levels in the atmosphere model. A survey on how monsoon rainfall and ISOs are captured in CESM is given in the SI. The details of the model experiments are also provided in the SI.

## Electronic supplementary material

Supplementary Information

## References

[1] Goswami B, Venugopal V, Sengupta D, Madhusoodanan M, Xavier PK (2006). Increasing trend of extreme rain events over india in a warming environment. Science.

[2] Allan RP, Soden BJ (2008). Atmospheric warming and the amplification of precipitation extremes. Science.

[3] Dash SK, Kulkarni MA, Mohanty UC, Prasad K (2009). Changes in the characteristics of rain events in India. J. Geophys. Res.-Atmos..

[4] Ajayamohan R, Merryfield WJ, Kharin VV (2010). Increasing trend of synoptic activity and its relationship with extreme rain events over central india. J. Climate.

[5] Karmakar N, Chakraborty A, Nanjundiah RS (2015). Decreasing intensity of monsoon low-frequency intraseasonal variability over India. Environ. Res. Lett..

[6] Zhou T, Zhang L, Li H (2008). Changes in global land monsoon area and total rainfall accumulation over the last half century. Geophys. Res. Lett..

[7] Bollasina MA, Ming Y, Ramaswamy V (2011). Anthropogenic aerosols and the weakening of the South Asian summer monsoon. Science.

[8] Ghosh S, Das D, Kao S-C, Ganguly AR (2012). Lack of uniform trends but increasing spatial variability in observed indian rainfall extremes. Nature Clim. Change.

[9] Turner AG, Annamalai H (2012). Climate change and the South Asian summer monsoon. Nature Clim. Change.

[10] Guhathakurta P, Rajeevan M (2008). Trends in the rainfall pattern over india. Int. J. Climatol..

[11] Fischer EM, Knutti R (2015). Anthropogenic contribution to global occurrence of heavy-precipitation and high-temperature extremes. Nature Clim. Change.

[12] Karmakar N, Chakraborty A, Nanjundiah RS (2017). Space? @ STime Evolution of the Low- and High-Frequency Intraseasonal Modes of the Indian Summer Monsoon. Mon. Wea. Rev..

[13] Karmakar, N., Chakraborty, A. & Nanjundiah, R. S. The spatio-temporal structures and role of low-and high-frequency intraseasonal modes in indian summer monsoon rainfall observed in trmm data. In *Proc. SPIE 9882, Remote Sensing and Modeling of the Atmosphere, Oceans, and Interactions VI*, 988227 (International Society for Optics and Photonics, 2016).

[14] Singh D, Tsiang M, Rajaratnam B, Diffenbaugh NS (2014). Observed changes in extreme wet and dry spells during the south asian summer monsoon season. Nature Clim. Change.

[15] Zveryaev I (2002). Interdecadal changes in the zonal wind and the intensity of intraseasonal oscillations during boreal summer Asian monsoon. Tellus A.

[16] Zuo Z (2012). Role of thermal condition over Asia in the weakening Asian summer monsoon under global warming background. J. Climate.

[17] Devaraju N, Bala G, Modak A (2015). Effects of large-scale deforestation on precipitation in the monsoon regions: Remote versus local effects. Proc. Natl. Acad. Sci. USA.

[18] Paul, S. *et al*. Weakening of indian summer monsoon rainfall due to changes in land use land cover. *Sci. Rep*. **6** (2016). 10.1038/srep32177 PMC4995379 27553384

[19] Ghil, M. *et al*. Advanced spectral methods for climatic time series. *Rev. Geophys*. **40**, 3-1–3-41, doi:10.1029/2000RG000092.1003. (2002)

[20] Rajeevan M, Bhate J, Kale J, Lal B (2006). High resolution daily gridded rainfall data for the indian region: Analysis of break and active monsoon spells. Curr. Sci..

[21] Lu, J., Vecchi, G. A. & Reichler, T. Expansion of the Hadley cell under global warming. *Geophys. Res. Lett*. **34** (2007).

[22] Vecchi GA, Soden BJ (2007). Global warming and the weakening of the tropical circulation. J. Climate.

[23] Ding Q, Wang B (2007). Intraseasonal teleconnection between the summer Eurasian wave train and the Indian Monsoon. J. Climate.

[24] Moorthi S, Arakawa A (1985). Baroclinic instability with cumulus heating. J. Atmos. Sci..

[25] Xie X, Wang B (1996). Low-frequency equatorial waves in vertically sheared zonal flow. Part II: Unstable waves. J. Atmos. Sci..

[26] Srinivasan J, Smith G (1996). The role of heat fluxes and moist static energy in tropical convergence zones. Mon. Wea. Rev..

[27] Chakraborty A, Nanjundiah RS, Srinivasan J (2014). Local and remote impacts of direct aerosol forcing on Asian monsoon. Int. J. Climatol..

[28] Hamada, A., Takayabu, Y. N., Liu, C. & Zipser, E. J. Weak linkage between the heaviest rainfall and tallest storms. *Nat. Commun*. **6** (2015). 10.1038/ncomms7213 PMC4346623 25708295

[29] Munemoto M, Tachibana Y (2012). The recent trend of increasing precipitation in Sahel and the associated inter-hemispheric dipole of global SST. Int. J. of Climatol..

[30] Seo K-H, Ok J, Son J-H, Cha D-H (2013). Assessing future changes in the East Asian summer monsoon using CMIP5 coupled models. J. of Climate.

[31] Abish B, Joseph P, Johannessen OM (2013). Weakening trend of the tropical easterly jet stream of the boreal summer monsoon season 1950–2009. J. Climate.

[32] Sharmila S, Joseph S, Sahai A, Abhilash S, Chattopadhyay R (2015). Future projection of Indian summer monsoon variability under climate change scenario: An assessment from CMIP5 climate models. Glob. Planet. Change.

[33] Vinoj V (2014). Short-term modulation of Indian summer monsoon rainfall by West Asian dust. Nature Geosci..

[34] Bhattacharya, A., Chakraborty, A. & Venugopal, V. Role of aerosols in modulating cloud properties during active–break cycle of Indian summer monsoon. *Climate Dyn*. 1–15 (2016).

[35] Kalnay E (1996). The NCEP/NCAR 40-year reanalysis project. Bulletin of the American meteorological Society.

[36] Krishnamurthy V, Shukla J (2007). Intraseasonal and seasonally persisting patterns of Indian monsoon rainfall. J. Climate.

[37] Rajeevan M, Gadgil S, Bhate J (2010). Active and break spells of the Indian summer monsoon. J. Earth. Syst. Sci..

[38] Hurrell JW (2013). The Community Earth System Model: A Framework for Collaborative Research. Bull. Amer. Meteor. Soc..

[39] Hurrell JW, Hack JJ, Shea D, Caron JM, Rosinski J (2008). A new sea surface temperature and sea ice boundary dataset for the Community Atmosphere Model. J. Climate.

