# Supplementary material for: Increased sporadic extremes decrease the intraseasonal variability in the Indian summer monsoon rainfall
Source: Sci Rep. 2017 Aug 10;7:7824. doi: 10.1038/s41598-017-07529-6 (PMC5552763; doi:10.1038/s41598-017-07529-6)
Supplement: Supplementary file 1 — Supplementary Information [file 41598_2017_7529_MOESM1_ESM.pdf]

Supplementary Information for ”Increased sporadic extremes decrease the intraseasonal variability in the Indian summer monsoon rainfall”

Nirupam Karmakar[1], Arindam Chakraborty[*,1,2], and Ravi S. Nanjundiah[1,2]

[1]*Centre for Atmospheric and Oceanic Sciences, Indian Institute of Science, Bangalore 560012, India.*

[2]*Divecha Center for Climate Change, Indian Institute of Science, Bangalore 560012, India.*

It is established that the intensity of the low-frequency intraseasonal oscillations (LF-ISO) observed in rainfall over India has reduced in the past few decades [1]. Associated with that, the percentage of extreme events in break phase increased in the backdrop of a sharp increase of total extreme events (within a season) over India. This leads us to the following question: Do the occurrences of more extreme events in break phase attenuating the variability observed in the LF-ISO scale? Extreme rainfall events are generally embedded in the large-scale ISO structures [1]. In this paper, we investigated ***whether the weakening of this association is a cause of the reduction in the LF-ISO intensity or not. In other words, we examined if the occurrence of more extreme events in break phase over CI causing the rainfall variability in LF-ISO scale lessened***. We used Community Earth System Model version 1.2 (CESM1.2) [2] (`http://www.cesm.ucar.edu/models/cesm1.2/`) to examine this hypothesis.

We performed a control experiment in the model with prescribed monthly varying cli-

matological sea surface temperature (SST) and then performed a simple experiment with implementing heating in the troposphere (top-heavy) during selected days on random grid-points in break phase over the central Indian (CI) region obtained from the control experiment. The additional atmospheric heating is expected to create a conducive environment for heavy rainfall in a very short timescale. There are several studies which used earlier versions of this model which implemented heating structure in the atmosphere to understand Madden-Julian Oscillation (MJO) and simulate its structure in a more realistic way [3, 4]. The response of the circulation over the Indian region due to El-Niño diabatic heating was also studied in a similar fashion by adding idealized heating profiles in some studies [5, 6]. The added heating in those studies were aimed to understand and simulate MJO or to address the effects of ENSO-related heating. Our goal here is different from those, as we want to generate extreme rainfall events on random points over CI for a day or so. But to understand how and where this heating should be added, requires a careful analysis from the observation of extreme events. Therefore, before going into the modeling experiments, we performed a thorough analysis on the extreme rainfall events in break and active phases of rainfall over CI region and the same is presented in the next section. This study lead us to design the experiment to understand role of extreme events in phases.

1 Indian monsoon in CESM and its earlier versions

There has been several studies which targeted understanding monsoon processes using CESM or its earlier versions (Community Climate System Model (CCSM)). Few studies used the Coupled Model Intercomparison Project 3 and 5 (CMIP3 and CMIP5; http://cmip-pcmdi.llnl.gov/) datasets, which included CCSM and CESM, analysing the behaviour of Asian monsoon in different models. It was concluded that the CMIP5 models perform better in simulating Asian monsoon than the CMIP3 models in an overall sense [7]. Importantly, they reported that CCSM4 was one of the best models in simulating the climatology and the climatological annual cycle. The El-Niño Southern Oscillation (ENSO)-monsoon relationship and boreal summer ISV were also captured reasonably

well in the model. It was also concluded in a study that CESM-CAM5 and CCSM4 both performed well in simulating the seasonal cycle of Indian and Australian monsoon [8]. However, the performance in simulating the ENSO-Indian monsoon relation was not impressive in CESM-CAM5. Monsoon-Indian Ocean Dipole (IOD) relationship was poorly simulated in almost all the CMIP models. Few studies investigated the boreal summer ISO in several CMIP5 models' historical runs [9]. They concluded that all the state-of-the-art models still have difficulties in simulating the monsoon ISV. CCSM4 captured the eastward propagations along the equator. The northward propagation over the Indian region is seen in CCSM4 but the amplitude near the equator is very less compared to observations. Also, the captured variance in ISO timescale is poorly simulated in this model. The nature of changes in the mean precipitation and extreme rainfall events over the Asian summer monsoon region in historical and future projection runs in numerous CMIP5 models including CCSM4 was also investigated it was found that there is an increase in precipitation over the north Asian summer monsoon region with a decrease in the southern part [10]. It is also shown that the percentile rainfall intensity over the Asian monsoon region is captured in CCSM4 model better than many other models. Although all the models systematically underestimate the precipitation intensity in heavy and heaviest rainfall thresholds, CCSM4 captures these rainfall intensities comparatively better than most of the models. Like most of the models, CCSM4 also resulted an increase in mean rainfall over the northern Asian monsoon region with increase in the intensity and frequency of the heaviest rainfall in the future projections. A moisture budget diagnostics also showed that the sharp increase in the water vapour in the atmosphere increases low-level moisture convergence, which essentially leads to an increase in the heaviest rainfall events.

There are numerous studies that used CESM or its earlier versions to investigate the modulation of Indian monsoon by black carbon [11] or aerosols [12, 13]. Therefore, from the above discussion, it can be concluded that although the simulations of Indian summer monsoon in CESM and CAM exhibit variations from observations, it is better than many of the present global climate models.

2 Characteristics of extreme events: Observations

Since we wanted to look into the nature of extreme events during monsoon season, all the analysis in this section are focused on June–September months. Also, we considered the CI region defined as in main text (16.5°S to 26.5°N and 74.5°E to 86.5°E). Our focus was to look into the nature of extreme rainfall events during active or break phases over CI.

2.1 Estimation of the number of grids showing extreme rainfall

The first goal was to identify how many points are typically seen over CI during active or break phases, where the rainfall exceeds a given threshold of extreme rainfall. To understand this, we used the 0.25° $\times$ 0.25° daily rainfall data from India Meteorological Department (IMD) (http://www.imd.gov.in/advertisements/20160219_advt_12.pdf) for the period 1979–2014 and regridded into model resolution, i.e., 0.9° $\times$ 1.25°. Regridding was done because we wanted to estimate the number of grids that show extreme rainfall at model resolution. Here we used the high resolution rainfall data instead of the 1° $\times$ 1° data used in the main text because of the regridding. However, the extremes were calculated here using the regridded rainfall data, which is implies that the spatial distribution and frequency of extremes are almost similar to those described in the main text. The period 1979–2014 was chosen because we also studied the heating profiles during the extremes using MERRA data (NASA's Modern-Era Retrospective Analysis for Research and Applications (MERRA) (obtained from Global Modeling and Assimilation Office (GMAO) and the GES DISC) dataset [14]) (https://gmao.gsfc.nasa.gov/reanalysis/MERRA/), which is given for the aforementioned period. For this resolution, we chose 90 mm/day as a threshold for extreme event at a grid point. This is because we have seen the typical threshold of extreme events over the CI region is about 90 mm/day [1]. However, the results do not change if we slightly change the threshold value. The next task was to define active/break phases of rainfall over this region based on this data. To do so, we extracted LF-ISO over the Indian region using multichannel singular spectrum analysis (MSSA) and determined active/break phases using the phase of the os-

cillatory signal [15]. While implementing MSSA, we have taken May–October data for a longer timeseries, but subsequent analyses done on June–September data.

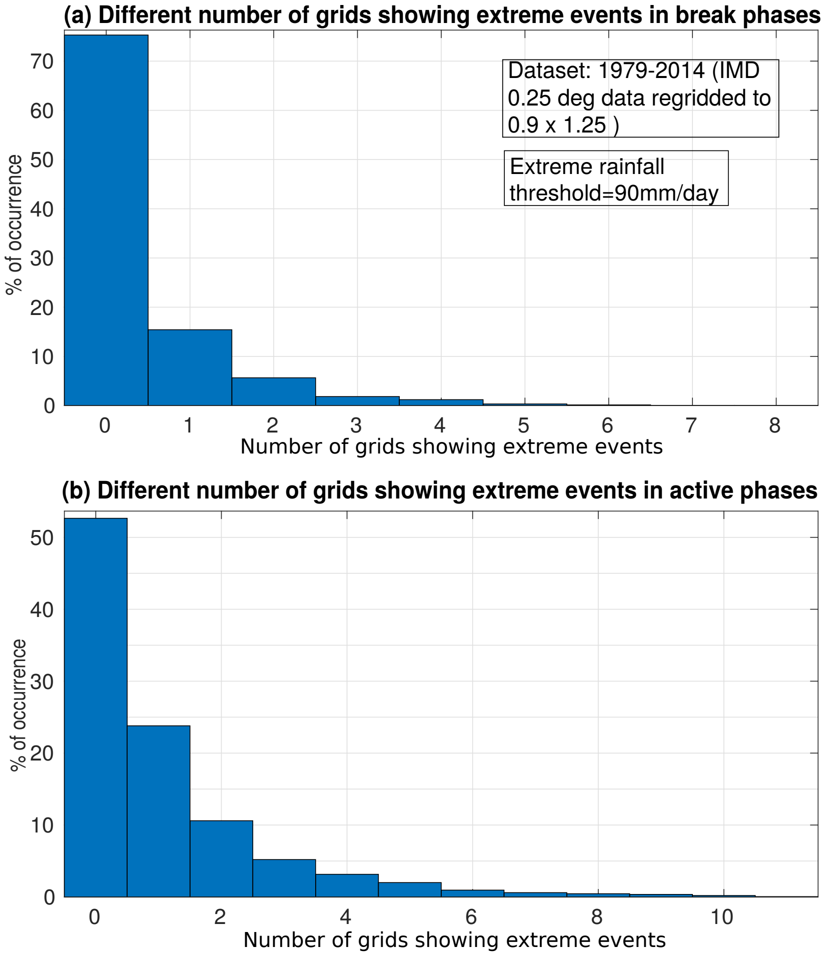

Figure S1: **Extreme rainfall variability as a function of grids and days during actives and breaks over CI. (a)** The variability of the number of occurrences with number of gridpoints having extreme rainfall during break phase over CI. $x - axis$ denotes the number of grids over which extreme rainfall is seen on a day. x=0 implies there is no extreme event. $y - axis$ denotes the percentage of the total break days. and **(b)** Same as (a) but for active phase. Figures are generated using MATLAB R2015a (https://in.mathworks.com/products/new_products/release2015a.html).

We calculated the number of grids over the CI region which show extreme rainfall during active or break phases in June-September months for each year. We observe that all the days in break or active phases may not have an extreme rainfall event. In fact, majority of the days do not have any extreme rainfall over CI. The histogram of the number of grids in the ISO phases are shown in Fig. S1. Fig. S1a shows the percentage of break days as a function of the number of grids with extreme rainfall. This suggests that almost 75%

of the break days do not have any extreme event over CI. Whereas, almost 16% of the break days have only one point over CI on which extreme rainfall occur. As the number of grid increases, the percentage of occurrence decreases sharply. This indicates that extreme rainfall events occur occasionally at very few places over the CI region during breaks. In other words, typically there are very few gridpoints over CI where extreme rainfall is seen during the 25% of the break days. Similar conclusions can be drawn for the active phase extreme events. However, the numbers are slightly different than that of the break phase. Almost 55% of active days do not have any point over CI where extreme rainfall is seen.

2.2 Diurnal variability of rainfall during extreme rainfall events

The next question needed to be addressed was: When does the peak in rainfall occur during an extreme event? or How is the diurnal cycle of rainfall during extreme rainfall events in break/active phases? To examine this, we needed a finer temporal resolution data. We use MERRA rainfall data for 1979–2014, which is available in hourly format in 0.5° × 0.66° spatial resolution. We regridded the data into 1.25° × 1.25° and also interpolated the hourly data into 3-hourly format to match it with the space-time resolution of the temperature tendency dataset in MERRA. The region of analysis remained the same (CI).

Since the data is of coarser resolution, we set 75 mm/day as the threshold for extreme rainfall over any grid over CI. We identified the grids where the extreme rainfall occurred during active and break phases. Then we examined the diurnal variation of rainfall within those grids on extreme rainfall days. Fig. S2 shows the variation within a day when extreme rainfall occurred in break or active phases. It shows that there exists a diurnal variation in rainfall in break phase extremes. After 10:30AM (UTC time; all the time mentioned are in UTC), the rainfall increases with time and the maximum of rainfall occurs at 7:30PM. There exists another peak at 4:30AM, but with lesser amplitude. Active phase extremes also show a similar pattern except that the afternoon peak is less pronounced than in break phase and rainfall occurs at almost same rate from 1:30PM–7:30PM. The 4:30AM maximum is also

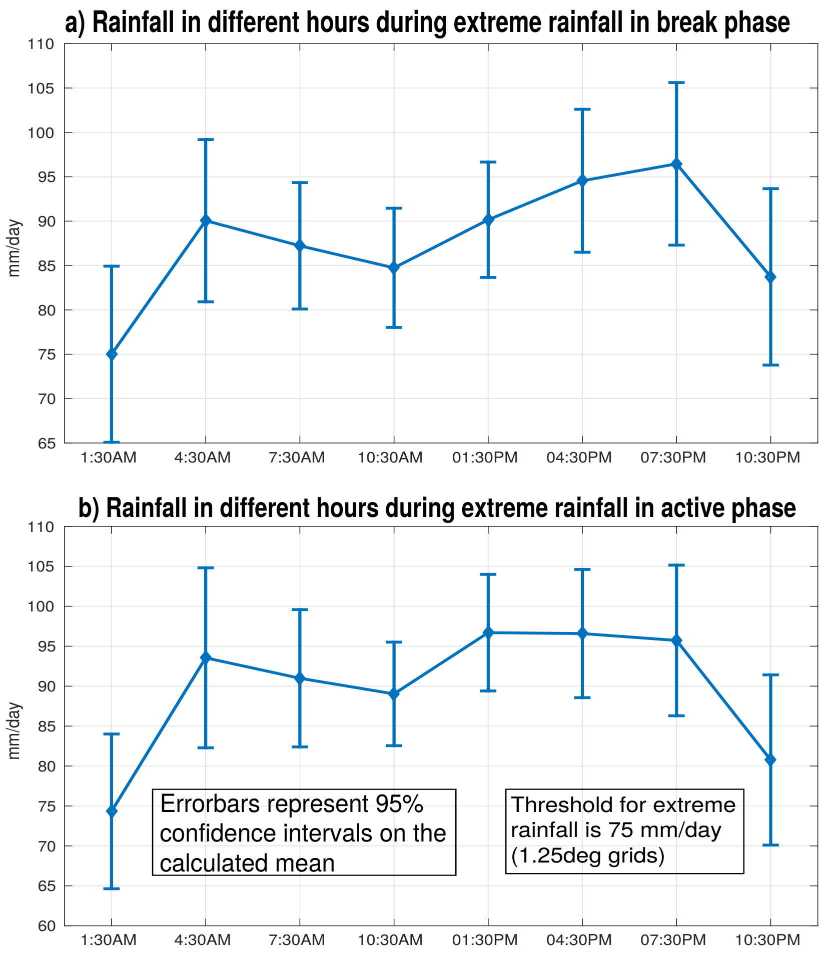

Figure S2: **Diurnal variation of rainfall during extremes in break and active phases over CI. (a)** Diurnal variation of rainfall during extreme events in break phases over CI and **(b)** Same as (a) but for active phases. $x - axis$ represents time given in UTC (Coordinated Universal Time). $y - axix$ is given in mm/day. Figures are generated using MATLAB R2015a (https://in.mathworks.com/products/new_products/release2015a.html).

present in active phase extremes. Therefore, the rainfall during the extreme events have a diurnal variability and is maximum in the 4:30–7:30PM. To confirm this, we checked with the TRMM 3-hourly rainfall data and found similar results, but the intensity of rainfall is slightly higher than in MERRA.

2.3 Heating structure during the extreme events

The vertical profile of the mean June–September CI averaged temperature tendency (MERRA reanalysis) is given in Fig. S3. On average, the mid-troposphere (600-350 hPa)

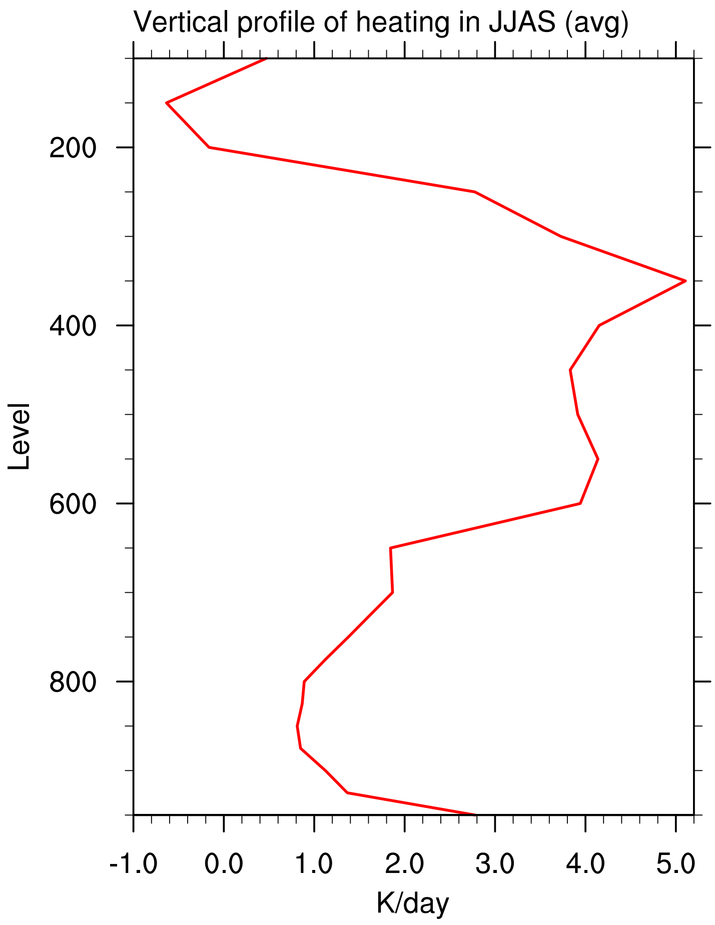

Figure S3: **June–September average heating profile over CI.** Vertical profile of heating (temperature tendency from physics) during June-September averaged over CI for 1979–2014. Unit is given in K/day. Data taken from MERRA reanalysis. Figures are generated using NCAR Command Language 6.3.0 (http://www.ncl.ucar.edu/).

shows heating during the monsoon season with a rate of almost 4–5 K/day. Our next goal is to see how the temperature tendency changes with the variation of rainfall on diurnal scale during the extreme events. To do so, we apply similar methodology to identify active/break phases and calculate the extreme rainfall grids. Then we look into the vertical structure of the temperature tendency at different hours of day over the grids with extremes, in both active and break phases. Fig. S4 shows that a top heavy profile is preferred during extreme events. Maximum heating can reach almost 100 K/day at particular instances at the 300–400 hPa level. The heating profile also shows a prominent diurnal variation. Maximum heating is seen between 4:30PM–7:30PM UTC and on average, the heating in the mid-troposphere is around 45–50 K/day. The rainfall also peaks at that time as we have seen.

It is also observed that the break phase extremes heating profile show sharp peaks in the middle troposphere compared to the active phase extremes. From this analysis an estimate of the diurnal variation of the heating associated with the extreme rainfall events during break or active phases is obtained.

2.4 Relationship between rainfall and tropospheric heating during extreme events

The final analysis done here was to understand how the heating profile changes with time if the time of maximum rainfall is given within a day over an extreme rainfall grid. In other words, is there any lead-lag relationship between the rainfall maximum and heating maximum during an extreme event? This also provided the exact amount of atmospheric heating just before and during the maximum rainfall during an extreme event. To understand this, we calculated the hours of maximum rainfall during the extreme events in active and break phases over CI. Then we arranged the atmospheric heating 3-hourly data according to the maximum rainfall hour for that day. For example, let us assume that at a grid point an extreme event occurred and maximum rainfall occurred at 4:30PM on that day. Then we have looked into the heating profiles on that day and how heating evolved before the maximum rainfall, and also what happened just after it. This is done to understand if we have an extreme rainfall at a particular point, then when is the preferred time of maximum heating. This is shown in Fig. S5. Time $T\ hour$ indicates the time of maximum rainfall. Heating profiles till $(T - 18)$ hours are shown, i.e., 18 hours before maximum rainfall. We also show $(T + 3)$ hour, which is 3 hours after the maximum rainfall, in the last panel. It is quite evident that the heating in the middle troposphere is maximum just before and during the maximum rainfall in extreme events. The average heating rate is as high as 78 K/day near 400 hPa level. With increase lead-time (with respect to hour of maximum rainfall) the heating rate decreases. Also, the heating rate reduces after the maximum rainfall occurs in a day.

It is observed that the maximum heating occurs within 600–350 hPa level. So, to visu-

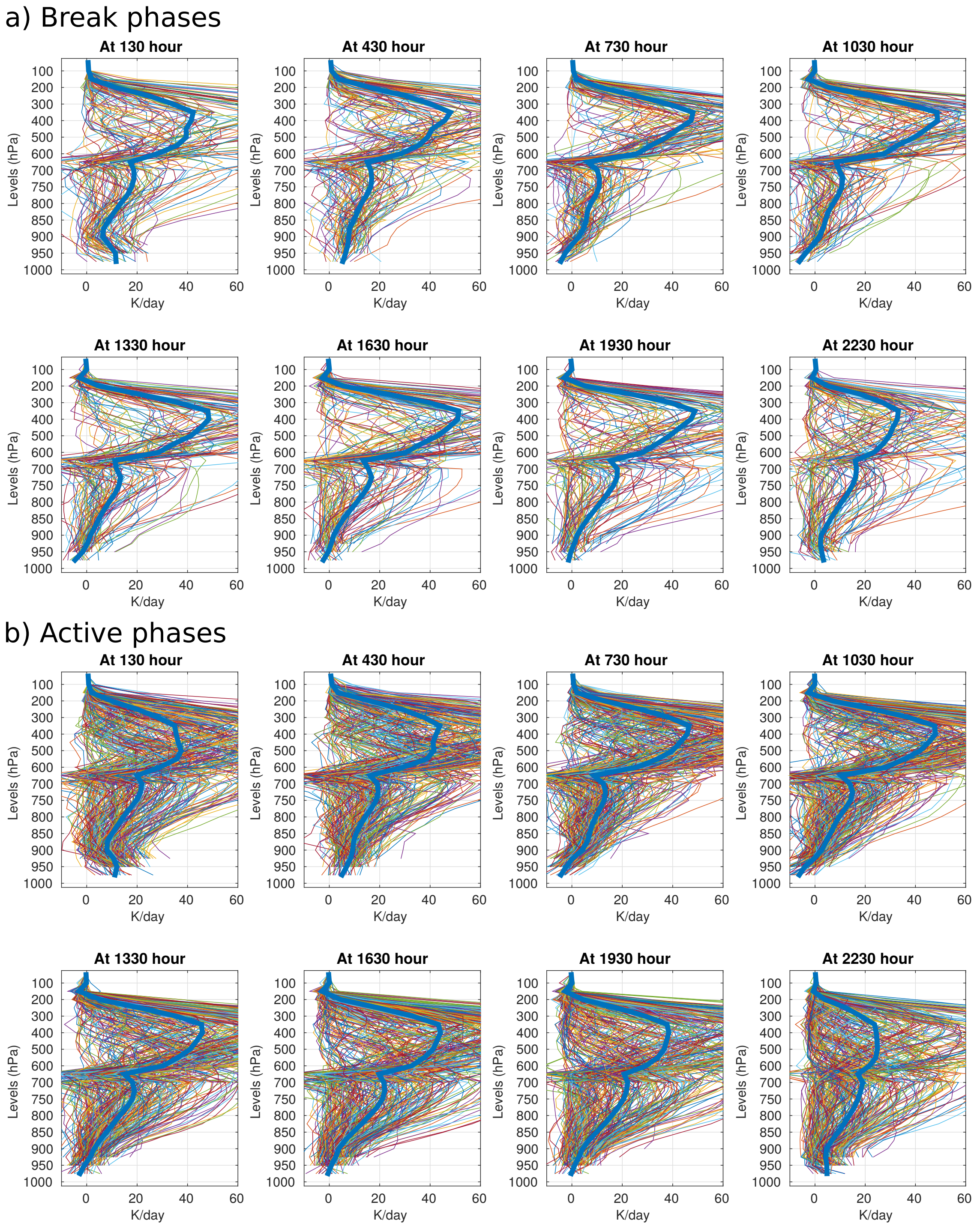

Figure S4: **Heating profile during extremes over CI during break and active phases.** **(a)** Diurnal variation of temperature tendency during extreme events in break phases over CI and **(b)** Same as (a) but for active phases. Thin lines indicate the profile in a single incident and the thick blue line is the average of all the samples. $x-axis$ represents heating and is given in K/day. $y-axix$ is vertical levels in hPa. Eight Fig. in each panel represents different time given in UTC. Figures are generated using MATLAB R2015a (https://in.mathworks.com/products/new_products/release2015a.html).

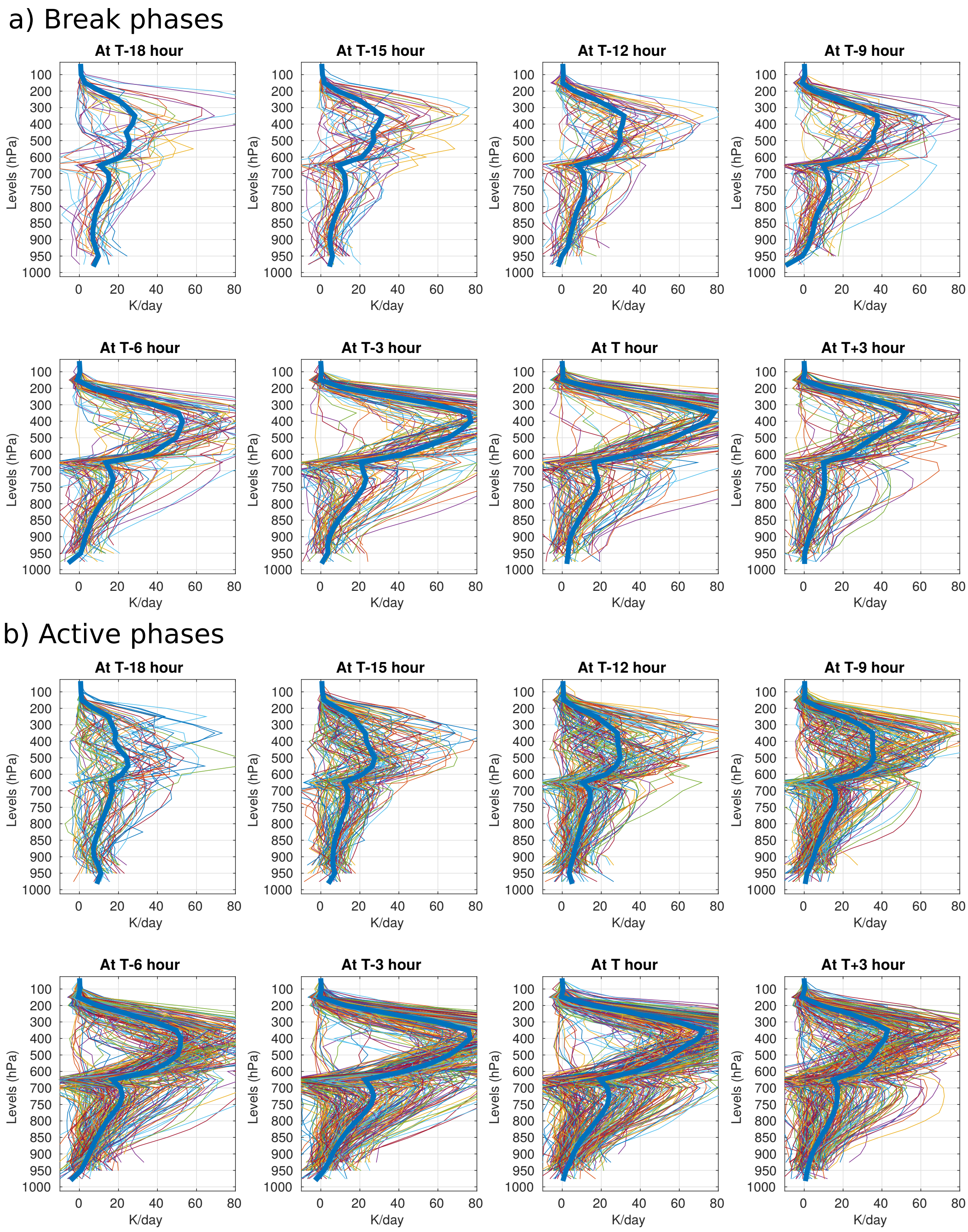

Figure S5: **Rainfall-heating profile relation during extremes over CI in break and active phases.** **(a)** Heating profiles before and after the time of maximum rainfall in a day during extreme events in break phases over CI. $T\ hour$ indicates the time of maximum rainfall. **(b)** Same as (a) but for active phases. Thin lines indicate the profile in a single incident and the thick blue line is the average of all the samples. $x-axis$ represents heating and is given in K/day. $y-axix$ is vertical levels in hPa. Figures are generated using MATLAB R2015a (https://in.mathworks.com/products/new_products/release2015a.html).

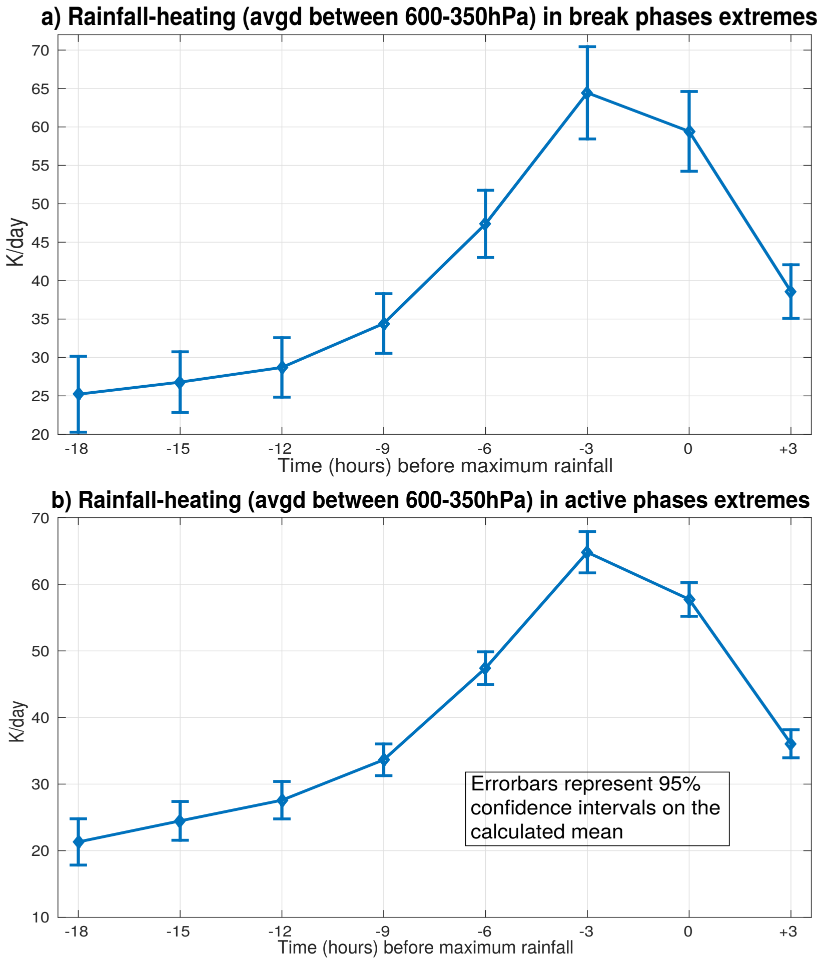

Figure S6: **Rainfall-heating profile (600–350 hPa) relation during extremes over CI in break and active phases.** **(a)** Heating rates averaged over 600–350 hPa as in Fig. S5 during break phase. **(b)** Same as (a) but for active phase. $x - axis$ represents time. 0 indicates the hour of maximum rainfall. $y - axix$ is given in K/day. Figures are generated using MATLAB R2015a (`https://in.mathworks.com/products/new_products/release2015a.html`).

alize the lead-lag relation in a better way, we have averaged the heating in the mentioned level and looked into the evolution of heating as a function of maximum rainfall within a day. This is shown in Fig. S6. It is clear that the heating rate averaged over 600-350 hPa level is maximum just before the maximum rainfall when an extreme event occurs. On an average, the heating rate averaged over 600–350 hPa is almost 65 K/day. During the maximum rainfall this rate drops to almost 58 K/day and after that it drops sharply. This characteristics of lead-lag between maximum rainfall and heating in the atmosphere is common in both active and break phases over CI.

In summary, in this section we understood the nature of the extreme events during the break and active phases: 1) How many days are typically observed during the active or break phases over CI when extreme rainfall events occur? 2) How many such gridpoints are observed in those days over CI? 3) What is the diurnal variability in rainfall during these extreme rainfall events? 4) What is the typical atmospheric heating structure associated with extreme rainfall events? and 5) How this heating profile changes with the time of maximum rainfall? These questions are needed to be addressed before performing the modeling experiments, as we will be prescribing additional heating into the atmosphere, which will lead to increased number of extreme events over the CI region during the break phase. This analysis provides us a pathway to define the nature of the heating to simulate the extreme events more realistically. In the next section, we provide the details of the experimental design.

3 Model experiment

The purpose of the modeling study is to understand the association of the extreme rainfall events with the ISO modes, essentially LF-ISO, over the Indian region. Our goal was to understand the vertical profile of heating during extreme rainfall events over the CI region during monsoon season, and how the characteristics of ISO change if there are more extreme events, especially during breaks. We performed two experiments: **1) A control experiment (CE),** and **2) A heating experiment (HE)**. We briefly describe the details of

control simulation and how heating is prescribed in the model using previous analysis.

3.1 Control experiment (CE)

Control experiment (CE) is the 11 years of model simulation without any changes from the default configuration (1st model year of this simulation is eliminated from any analysis considering a spin-up period).

3.2 Heating experiment (HE)

With the understanding from previous observational analysis on the characteristics of extreme rainfall events and the associated heating structure, in this section we discuss how heating is prescribed in the model. The basic idea was to generate heating over some random grid points in the CI region during June-September months, preferentially during the break periods, which would produce conducive environment for short spatial and temporal scale extreme rainfall events.

3.2.1 Prescription of heating

It is now clear that extreme rainfall events are observed at only few grid points (may be 1 or 2) over CI region. If we take only break phase extremes, then almost 75% of the break days do not have any grid with extreme rainfall. Thus, in our experiment, we heated only 1 or 2 points over the CI region on almost 25% of the break days (only over land region). The break days over CI are defined by the LF-ISO obtained from the CE, averaged over the CI region. The definition of the break days are similar to how it is defined in [15]. Also, we have heated only June–September month break days. So, typically there would be around 10–12 days (break days in the CE) in a season when heating is prescribed. These days are chosen randomly from the set of all CE break-days in a season using a uniform random number generator. In the next step, we choose gridpoints over CI region where heating is to prescribed, again using a uniform random number generator. We set the choice of random number such that the heating is prescribed typically at 1 or 2 grid points over CI. So, if we

look into a random day of heating, spatially it is like Fig. S7a.

The model may have its own extreme rainfall events over CI region. However, we imposed additional heating in the model to produce favourable environment to create more extreme rainfall events during break phase. The increased number is more or less equal to the number of extremes seen in observation. Therefore, it is expected that the HE will have more number of extremes over CI as compared to the CE (approximately 15–20 additional extreme events per season, considering the fact that heating is prescribed on 10–12 days on 1–2 points over CI). Now the most important step is to define the heating structure. Based on the observational study, we get an idea of the vertical profile of total heating in the atmosphere before and after few hours of maximum rainfall in an extreme event. It is observed that the maximum rainfall during extremes in break phase occurs at 4:30PM–7:30PM (UTC) hours. Associated with that, maximum heating is seen concurrently or just before it (see Fig. S5a and S6a). Based on this, we define the heating structure as given in Fig. S7b. Assume $F_z(k)$, given in K/day, is the amount of heating at vertical level k. The $F_z(k)$ is given by the following equation:

$$F_z(k) = \begin{cases} 0 \quad \text{for } k \in (0,150) \\ A_1 sin(\pi Z_1(k)) \times \exp(-b_1 Z_1(k)^4)) \quad \text{for } k \in \ (150,500) \\ 20 + (k-500)/75 \quad \text{for } k \in \ (500,650) \\ A_2 sin(\pi Z_2(k)) \times \exp(-b_2 Z_2(k)^{10})) \quad \text{for } k \in \ (650,1000) \end{cases} \tag{1}$$

where, Z_1=$(k-150)/(1100-150))$, Z_2=$(k-150)/(1000-k))$ and k is given in hPa; $A_1 = 138.24$, $b_1 = 65$, $A_2 = 30.24$ and $b_2 = 3$.

This kind of heating profile (F_z) is observed during and just before the maximum rainfall during an extreme rainfall event (Fig. S5a). The above equation matches the observations quite realistically.

Again, we have seen that the heating structure does not remain the same for the entire day. It is highest during and before the maximum rainfall and shows weaker signal during

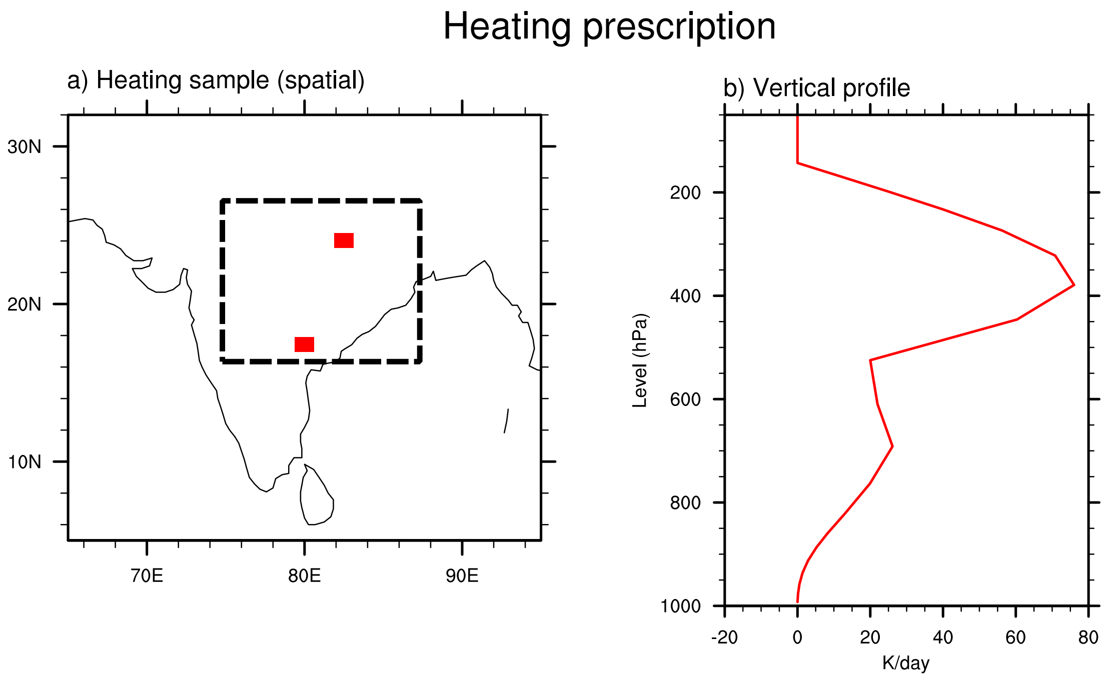

Figure S7: **Sample of prescribed heating over CI and heating profile.** **(a)** Sample of heating over CI region. The two red points are heated on this random day. The dotted box indicates the region where random gridpoints are chosen to heat. **(b)** Vertical structure of added heating in the atmosphere. Unit is in K/day. Figures are generated using NCAR Command Language 6.3.0 (http://www.ncl.ucar.edu/).

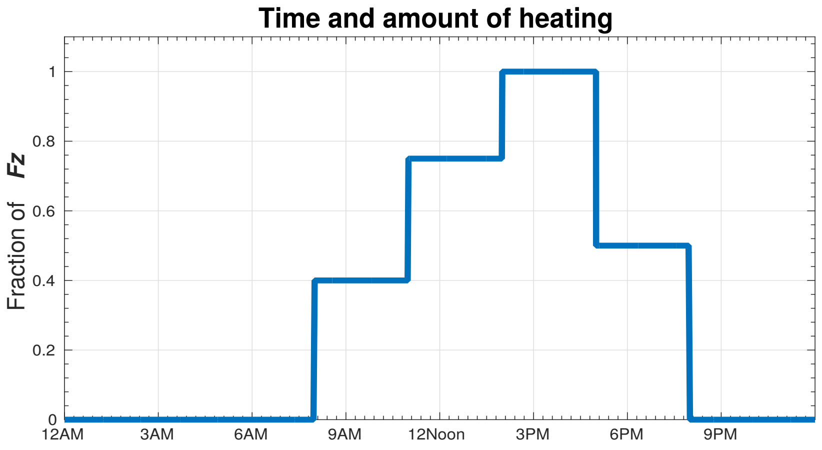

Figure S8: **Diurnal variation of prescribed heating.** Distribution of heating during a day. Time is given in UTC. Figures are generated using MATLAB R2015a (https://in.mathworks.com/products/new_products/release2015a.html).

rest of the day. Therefore, according to the observation, we have heated the atmosphere only 12 hours of the day, with varying amplitude. The diurnal distribution of heating is given in Fig. S8. It implies that the imposed heating is maximum (equals to F_z) during 2–5PM UTC, which matches Fig. S5a. The heating is provided during 8AM–8PM (UTC) over a particular grid point on a break day obtained from CE. In summary, the heating prescription is given in Table 1.

Table 1: Prescription of heating in the model

Parameter	Prescription	Quantity and Source
Days of heating	Randomly chosen break days (CE)	Roughly 10–12 days, Fig. S1a
Number of gridpoints to heat	Randomly chosen land points over CI	1 or 2 points, Fig. S1a and S7a
Time of heating in a day	12 hours	8AM–8PM, Fig. S5a, S6a and S8
Amount of heating	F_z in equation 1	Fig. S5a and S7b

3.2.2 Model simulation with imposed heating

We started the model HE every June 1st, using the restart files from the CE. Each simulation, then, continues for 5 months and ends on October 31st. Similar to CE, HE also was done for 10 years. These short simulations were necessary because after the heating was imposed in the model, the HE deviated from the CE and if we integrated long enough, the oscillatory signals might go entirely out-of-phase in two simulations. Basically, the HE and the CE are identical to the day of first heating from June 1st, every year. After the first heating event occurs over CI, the two simulations may behave differently.

For example, in the first year of HE, the first instance of heating is observed on June, 20 (a break day in CE). In Fig. S9a–c, the daily rainfall pattern in CE is shown for 20–22 June (day1–day3). Convective bands over the equatorial Indian Ocean and the north-eastern Indian region can be seen. In Fig. S9d–f, we have shown the difference in rainfall patterns between the HE and the CE for the same days. Quite evidently, the imposed heating over two random points over CI has produced excessive amount of rainfall over these locations in the HE (day1). On the next day, one of the location (southern one) dissipated and the other (northern one) strengthened. This could be because of the moisture supply or the presence

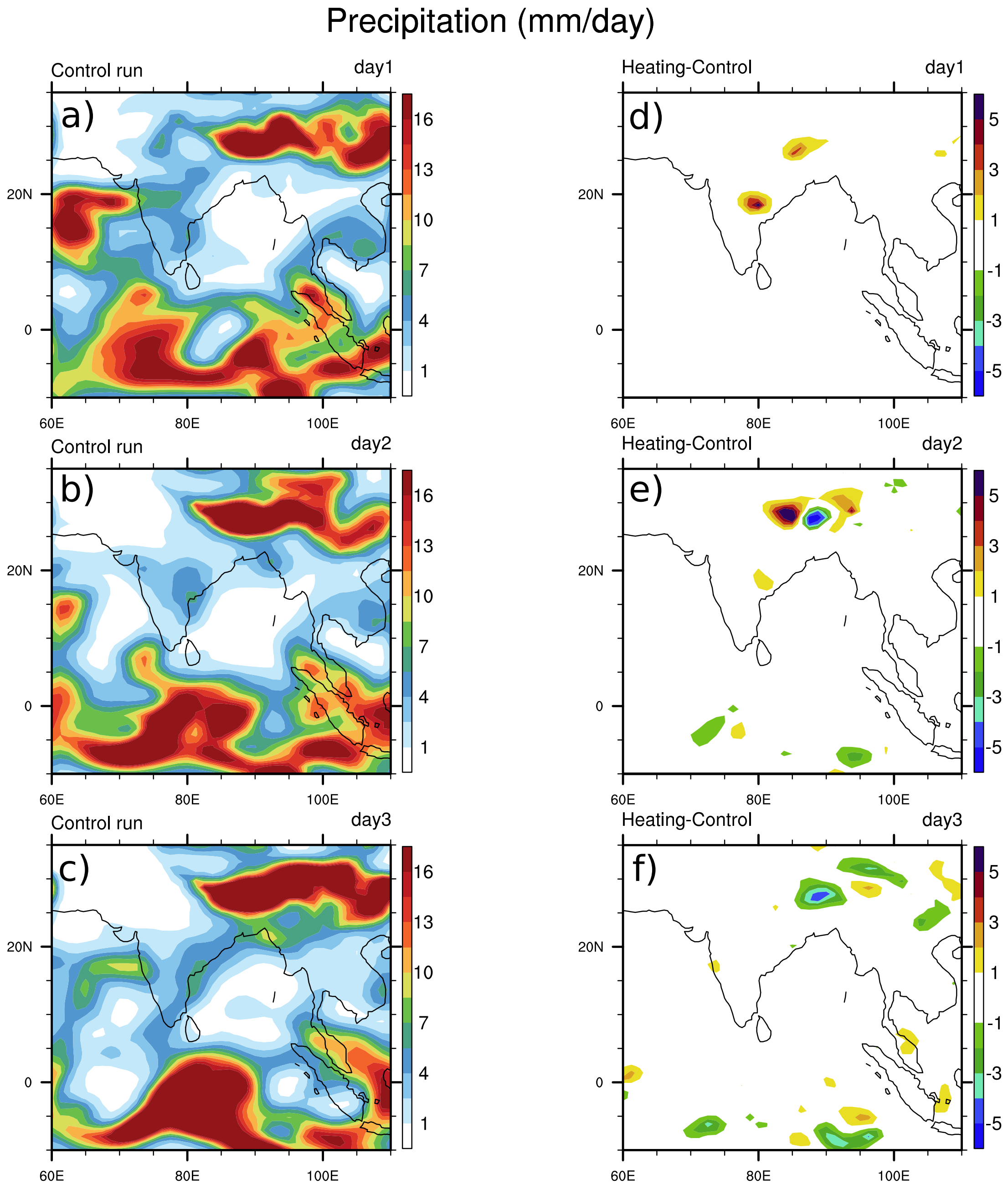

Figure S9: **Snapshots of rainfall after heating implemented.** Snapshots of daily rainfall patterns in the model CE during **(a)** Day 1, **(b)** Day 2, **(c)** Day 3 after the first implementation of heating over random CI gridpoints in the HE (20–22 June, 1st model year). **(d)–(f)** Difference in rainfall between the HE and CE during these days, respectively. Units are in mm/day. Figures are generated using NCAR Command Language 6.3.0 (http://www.ncl.ucar.edu/).

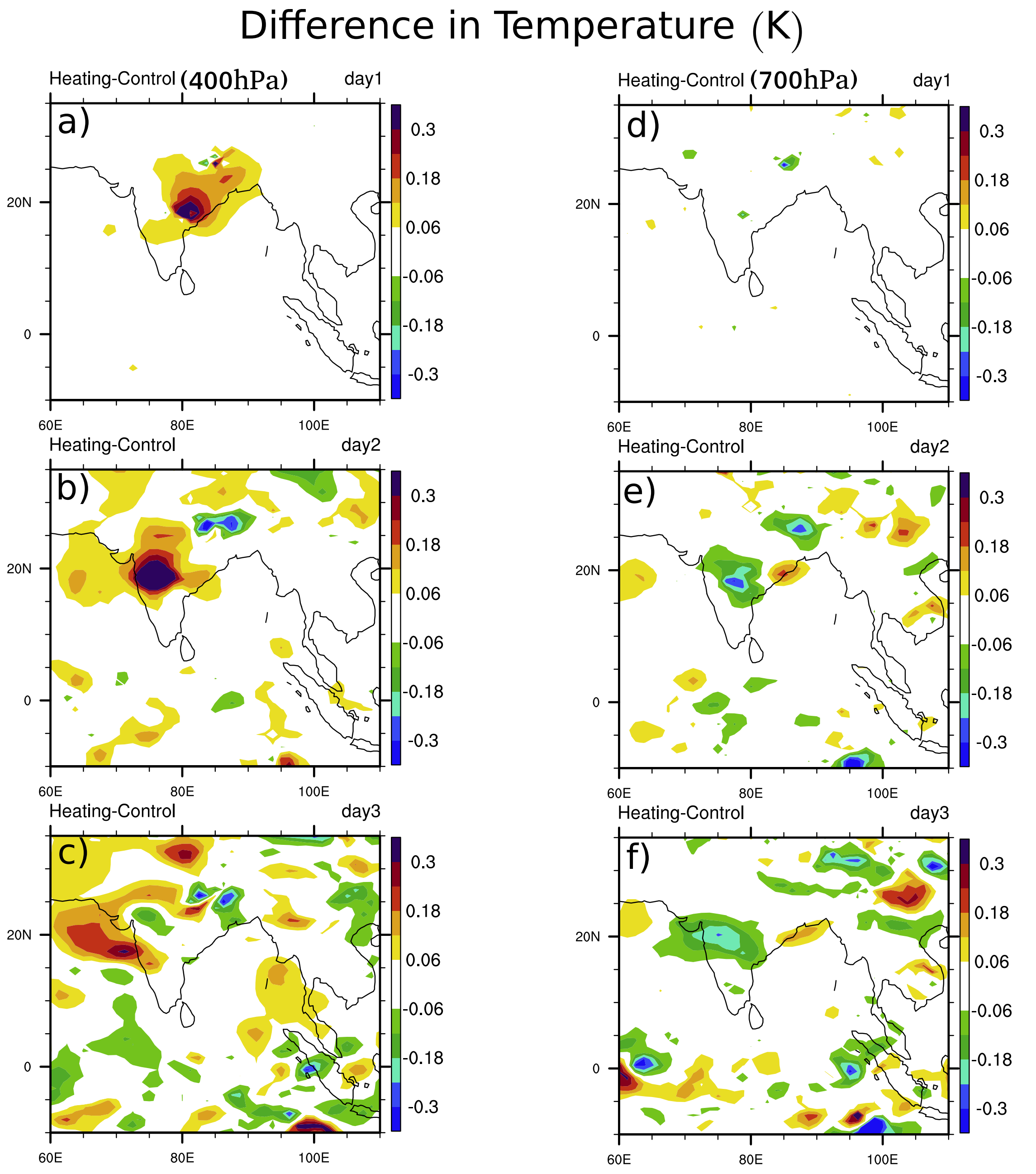

Figure S10: **Snapshots of atmospheric temperature after heating implemented.** Same as Fig. S9d–f, but for **(a)–(c)** 400hPa air temperature and **(d)–(f)** 700hPa air temperature. Units are in K. Figures are generated using NCAR Command Language 6.3.0 (`http://www.ncl.ucar.edu/`).

of the large-scale feature over north-eastern India. On the day3, the heavy rainfall spots disappeared from all the locations. But the imposed heating initiated a difference between the control and the HE, which gradually grows as time increases. This implies that although the imposed heating produced a short space- and time-scale heavy rainfall event, the HE and the CE LF-ISO phases may have a phase difference after the first instance of heating, which grows with time. Therefore, prescribing atmospheric heating only on break phase of the CE demands further attention. We have addressed this issue in the next section.

In Fig. S10a–c, we have shown the difference in air temperature at 400hPa level between the HE and the CE for the same dates (day1–day3). Quite evidently, the temperature at this level has increased after heating over the two locations in CI (day1). The southern point moved westward and gets strengthened in day2. After that it is dissipated over the Arabian Sea. However, the northern point does not show any strengthening or propagation after day1. The lower troposphere (700hPa) shows a negative difference, possibly because of advective cooling associated with an ascending movement of air, over the two points, which grows gradually with time (Fig. S10d–f). The temperature fields also show the gradual increase in the difference between the two simulations after the first instance of heating.

Examining the distribution of rainfall (Figure S11), it is observed that although the amount of very less rainfall (1–5 mm/day) has increased in the heating experiment (HE), the amount of moderate rainfall (5–25 mm/day) has decreased as compared to the control experiment (CE). Also, there is an increase in the higher amount of rainfall (more than 25 mm/day), which is expected. Looking into the tail of the distributions (Figure S11d), it can be concluded that the number of occurrences of extreme rainfall in the model has increased in the HE as compared to the CE. This was the goal of the modeling experiments and we intended to do so by prescribing atmospheric heating. Reduction in the moderate rainfall events is also documented in observations by [16]. We note here that it is a known fact that most of the global climate models, including CESM, do not produce extreme rainfall events with comparable magnitude as in observation [10].

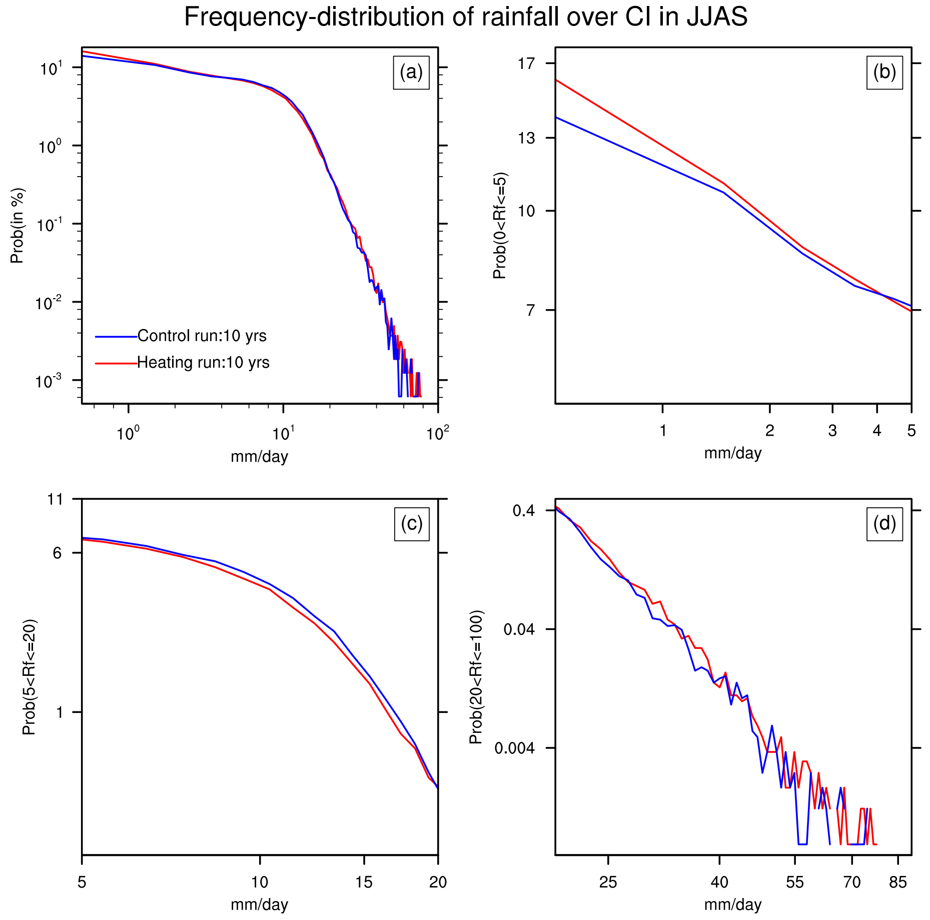

Figure S11: **(a)** Frequency distribution of rainfall over the CI region during June–September for the HE and the CE. For better understanding, we have zoomed this figure into **(b)** 1-5 mm/day, **(c)** 5–25 mm/day and **(d)** more than 25 mm/day.

4 Further analysis on model LF-ISO and distribution of extremes within active-break cycle

If we average the rainfall anomaly over the CI region for both the simulations, then for a particular year they would be identical until the first day of heating. This is shown in Fig. S12. An an example, after 2nd June of the 5th year, the HE deviated from the CE due to prescription of heating. LF-ISO averaged over CI also shows differences in both phase and amplitude in the two simulations. This is quite natural, as the heating prescribed over the region changed the rainfall patterns and as time increases the difference is expected to increase. This is the reason we have restricted the heating simulations to integrate for only

one season (June–October; while heating is done upto September) every year. We have also identified the active and break phases over CI in both the simulations. In Fig. S13a, we have shown the active days in both CE and HE for all the years. It is observed that the total number of active days has decreased in the HE by almost 7%. Also, the occurrences of moderate and very long active phase are less in the HE as compared to the CE (Fig. S13c). This indicates that the active phase in the HE tend to be of shorter length than in CE. Compared to this, the total number of break days has increased by 9.22% in the HE, with an increase in the occurrences of moderate and very long breaks (Fig. S13b and d). This leads to the fact that the length of the active and break phases are modified in the HE. It is seen that the first break or active phase in both the simulations starts almost in similar dates each year, which is expected. But in the 10th year the only break phase in CE occurred in the end of July, whereas, the first break of HE started during the end of June. This happened because, since we are filtering out the ISO in both simulations separately, in HE the rainfall alteration after end of July made the active-break cycle somewhat different for the entire season. Now, since the active-break phases are defined based on a mathematical equation, we get a break in heating before the break in control. However, the second break in this year of HE matches with that of the CE.

We note that the from Fig. S13, it is clear that if we prescribe heating based on the break days of CE, the HE would deviate from the CE just after the first day of heating. So, the breaks/actives in control and heating simulations may not match. Which implies that the target of heating in the breaks may not be achieved at 100% success rate. Some of the heating days will fall in active phase in the HE. To check this, we have calculated the percentage of days in each phase that are heated in the HE (Fig. S14a). It is observed that more than 12% of the break days are heated. Compared to that, less than 9% of active days are heated. Also, some heating occurred in break–active transition phase. We also calculated the percentage of the total number of heating days that fall in different phases in the HE (Fig. S14b). It is clearly seen that the majority (43%) of the heating days fall within break phase of the HE. 33% of the the heating days are in active phase. Therefore, since our target is to create extreme events, with majority occurring during break phases, we are

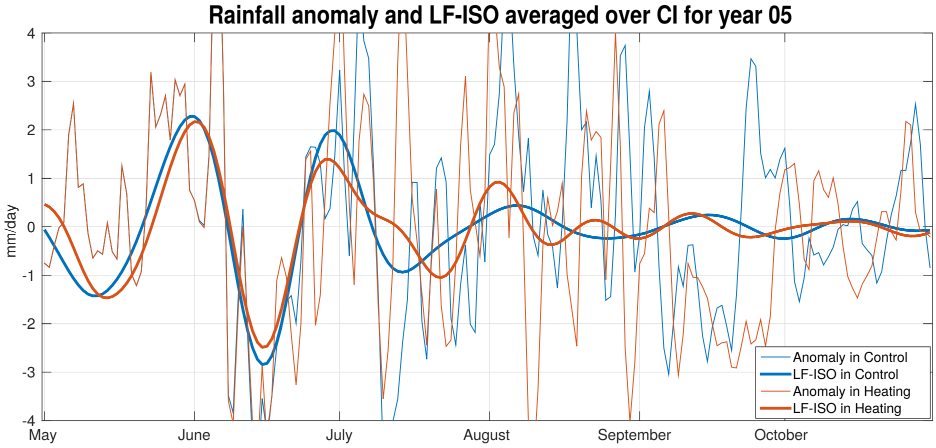

Figure S12: **CI averaged rainfall anomalies, and LF-ISO from the heating and the control experiments.** Rainfall anomalies averaged over the CI region for the CE and the HE for 5th year, taken as an example, are shown in thin lines. In thick lines the LF-ISO anomaly averaged over CI is shown for the same year. Figures are generated using MATLAB R2015a (`https://in.mathworks.com/products/new_products/release2015a.html`).

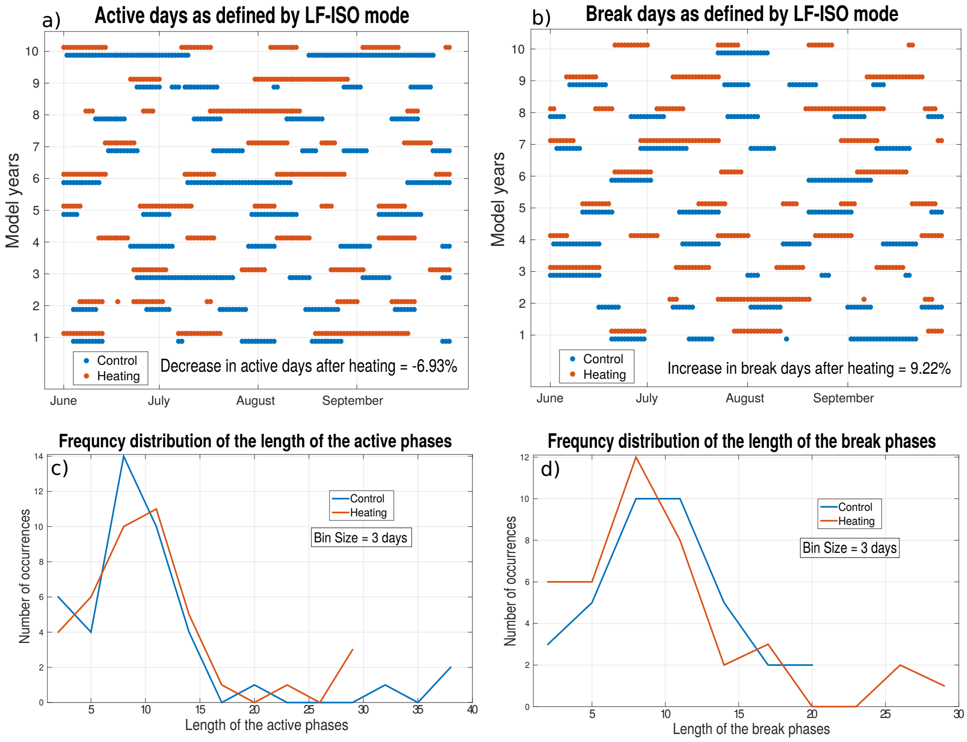

Figure S13: **Active and break days in the control and the heating experiments.** **(a)** Active days during June–September identified using LF-ISO mode from both the CE and the HE for 10 years. **(b)** Same as **(a)** but for break days. **(c)** Frequency of occurrences, in actual numbers, of active phase as a function of number of days for both 10 years of the CE and the HE. **(d)** Same as **(c)**, but for break phase. Figures are generated using MATLAB R2015a (`https://in.mathworks.com/products/new_products/release2015a.html`).

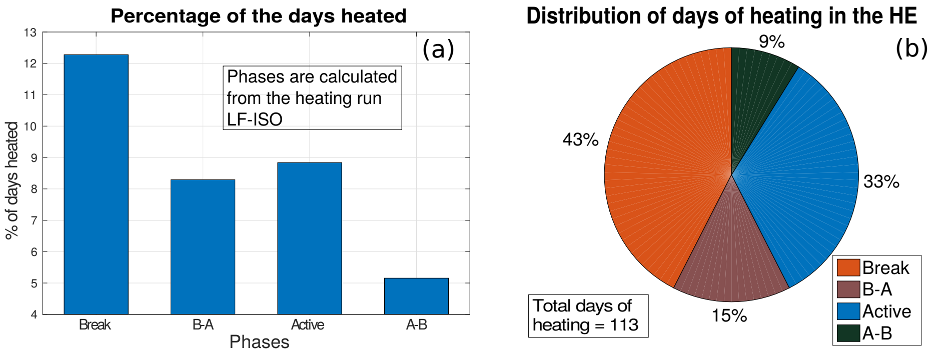

Figure S14: **Distribution of heating days in different phases in the heating experiment.** **(a)** Percentage of days in each phase that are heated in the model HE. **(b)** Percentage of the total heating days that fall in different phases in the HE. B–A and A–B indicate break–active and active–break transition phases. Figures are generated using MATLAB R2015a (`https://in.mathworks.com/products/new_products/release2015a.html`).

successful in it. This mimics the observed scenario of increasing extreme events, primarily in break phase, in recent decades.

5 Change in active phase rainfall

We found that the rainfall accumulated during the active phase has decreased significantly over the Indian region in both observations and model (Fig. S15). However, the changes over CI are more prominent in the observations. Model rainfall over the BoB and AS has also decreased in HE in the active phase. We also observe a increase in active phase rainfall over the equatorial Indian Ocean region. These changes are very similar to the changes in the seasonal mean.

6 Vertical moist stability analysis

Vertical moist stability (VMS) [17] of an atmospheric column is defined by the following equation:

$$VMS = MSE_{top} - MSE_{bot} \tag{2}$$

where, MSE_{top} and MSE_{bot} are defined as the column integrated moist static energy

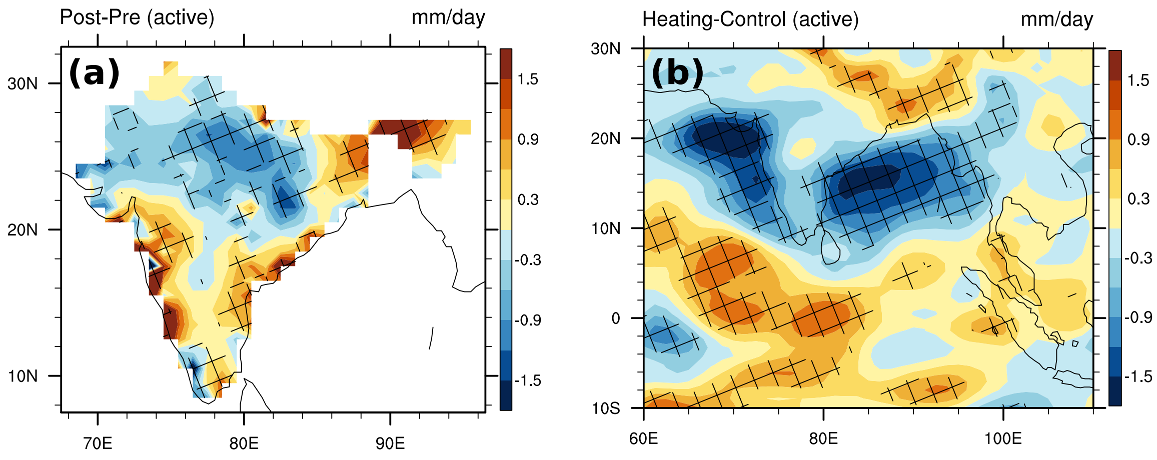

Figure S15: **Change in active phase rainfall.** Change in active phase total rainfall in **(a)** Observations (post80−pre80) and **(b)** Model (HE−CE), during JJAS months. Units are in mm/day. Hatched regions indicate places where the changes are significant at 10% level (using t-test). Figures are generated using NCAR Command Language 6.3.0 (http://www.ncl.ucar.edu/).

(MSE) of the upper and lower atmosphere, respectively, and they are defined as:

$$MSE_{top} = \frac{1}{(P_{mid} - P_{top})} \int_{P_{top}}^{P_{mid}} M dp$$

$$MSE_{bot} = \frac{1}{(P_{sfc} - P_{mid})} \int_{P_{mid}}^{P_{sfc}} M dp$$

Here, M is defined as the MSE of the of the atmosphere at any particular level. M is given as:

$$M = C_p T + gZ + L_c q \tag{3}$$

where,

C_p = 1005 J kg^{-1} K^{-1}, Specific heat of air at constant pressure.

L_c = 2.5 × 10^6 J kg^{-1}, Latent heat of condensation for water.

g = 9.81 m s^{-2}, Acceleration due to gravity.

T = Air temperature in Kelvin (K).

q = Specific humidity of air in kg kg^{-1}.

Z = Geopotential Height in meters (m).

P_{sfc} is the surface pressure, P_{mid} = 450 hPa and P_{top} = 100 hPa.

Similar to VMS, we also look into the vertical dry-static stability (VDS) of an atmospheric column, which is same as VMS except here we exclude the moisture term. VDS is defined as:

$$VDS = DSE_{top} - DSE_{bot} \tag{4}$$

where, DSE_{top} and DSE_{bot} are defined as the column integrated dry static energy (DSE) of the upper and lower atmosphere, respectively, and they are defined as:

$$DSE_{top} = \frac{1}{(P_{mid} - P_{top})} \int_{P_{top}}^{P_{mid}} Ddp$$

$$DSE_{bot} = \frac{1}{(P_{sfc} - P_{mid})} \int_{P_{mid}}^{P_{sfc}} Ddp$$

Here, D is defined as the DSE of the of the atmosphere at any particular level. D is given as:

$$D = C_pT + gZ \tag{5}$$

It is established that VMS can determine the location of deep clouds over any location. However, VMS below a threshold is a necessary condition and not sufficient for the existence of deep convective clouds [18, 19]. Role of VMS in formation of deep clouds was studied over the Pacific region [20] and also over Indian region [21]. Difference between the VMS and VDS can provide the importance of moisture term in the stability analysis.

In Figure S16a, June–September daily averaged VMS for the CE is shown. Typically, VMS is more over the oceanic regions compared to that over the land regions with values

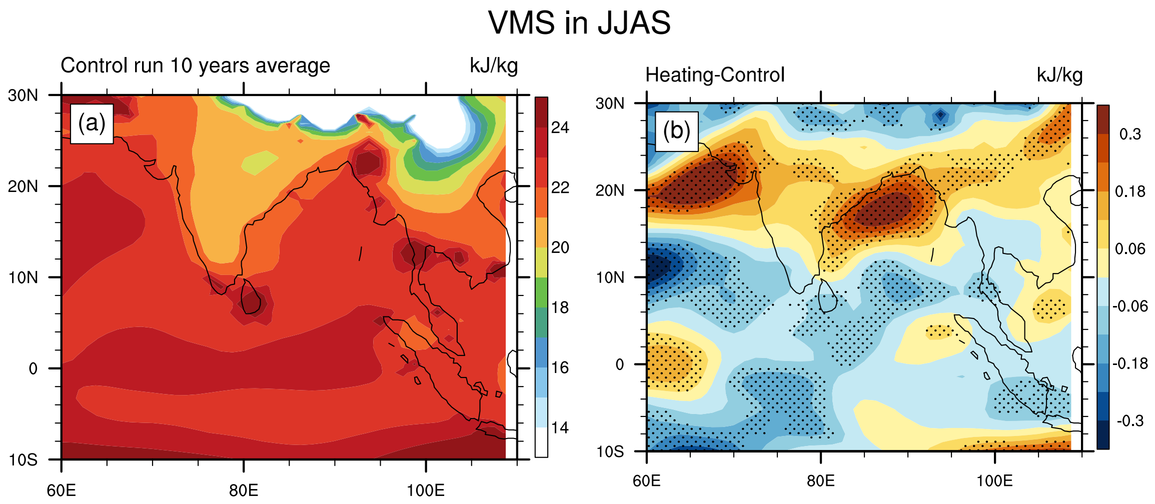

Figure S16: **Spatial distribution of VMS in the control and the heating experiments.** **(a)** June-September averaged VMS in 10 years of the CE. **(b)** Difference in June–September averaged VMS between the HE and the CE. The stippled regions indicate the regions where the difference is significant at 10% level (using paired t-test). Units are in kJ/kg. Figures are generated using NCAR Command Language 6.3.0 (http://www.ncl.ucar.edu/).

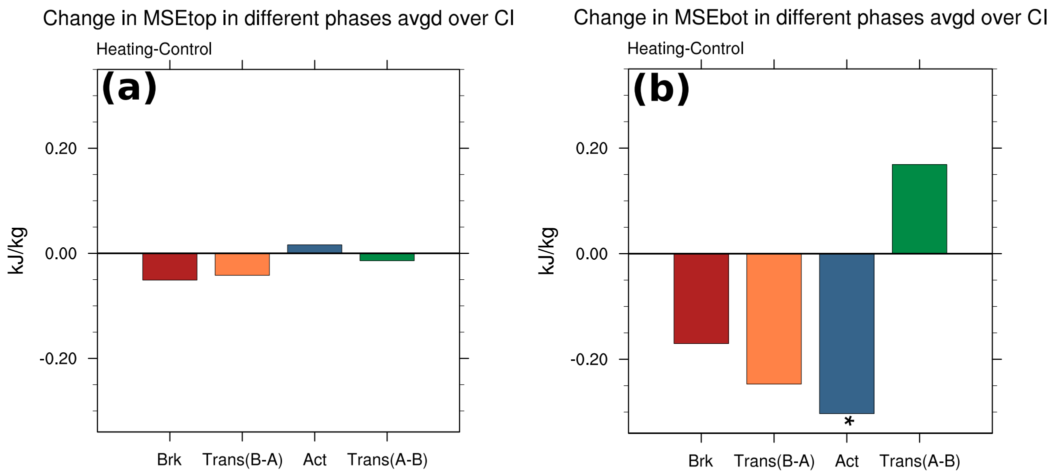

Figure S17: **Change in MSE over CI in different phases.** Change in vertically integrated MSE of the **(a)** upper troposphere and **(b)** lower troposphere between the HE and the CE. Different phases are described as Break, Transition (Break–Active), Active, and Transition (Active–Break), respectively. The asterisks indicate that the differences are significant at 10% level (using paired t-test). Units are in kJ/kg. Figures are generated using NCAR Command Language 6.3.0 (http://www.ncl.ucar.edu/).

of nearly 20 kJ/kg over CI. In Figure S16b the change in June–September daily averaged VMS in the HE compared to the CE is shown. It is evident that the VMS has increased over the CI region, the northern BoB and the AS. These are the regions where the June–September rainfall has decreased in the HE. Therefore, the reduction in rainfall over these regions is associated with the increase in VMS.

In the main text, the change in VMS in different phases in the HE and the CE is shown. A point to be noted here that different phases in the two simulations may have different lengths. It is evident that that active phase VMS over CI has increased significantly in the HE. Also, there is a gradual increase in VMS from break to active phase. There is a decrease in VMS in active–break transition phase. If we look into MSE of the upper and lower troposphere separately, it is clear that the increase in VMS is associated with a reduction of MSE in the lower troposphere (S17a and b). This clearly hints towards the role of moisture in the lower troposphere in the reduction of VMS in the HE. Also, it can be observed that break phase upper level MSE has decreased in the HE. This is possibly related to the less moisture supply to the upper levels due to larger scale downdrafts associated with increased number of small scale extreme events in break phase. We also observe that MSE of lower layer increased in HE in active–break transition phase. This is related to the increase in the near surface temperature due to less amount of rainfall in active phases.

The role of moisture in reduction of VMS is confirmed by Figure S18, where we have shown the change in vertical dry-static stability (VDS) and dry static energy (DSE) of upper and lower troposphere. VDS and DSE are calculated in a similar way as VMS and MSE, except removing the moisture term from the MSE equation. The difference in VMS and VDS will highlight the significance of the moisture term. The decrease in VMS in active–break phase is related to the increase in dry-static energy in lower layer (Figure S18c). It is quite evident that the change in VDS in different phases of LF-ISO, especially, break-active transition and active phases, is negligible. This indicates that the major change in stability of the atmosphere comes from the moisture term. It is the reduction in moisture supply, mainly in the lower atmosphere, that causes the increased atmospheric stability. To confirm this, we examined the zonal moisture flux at 70°E (over the Arabian Sea) at

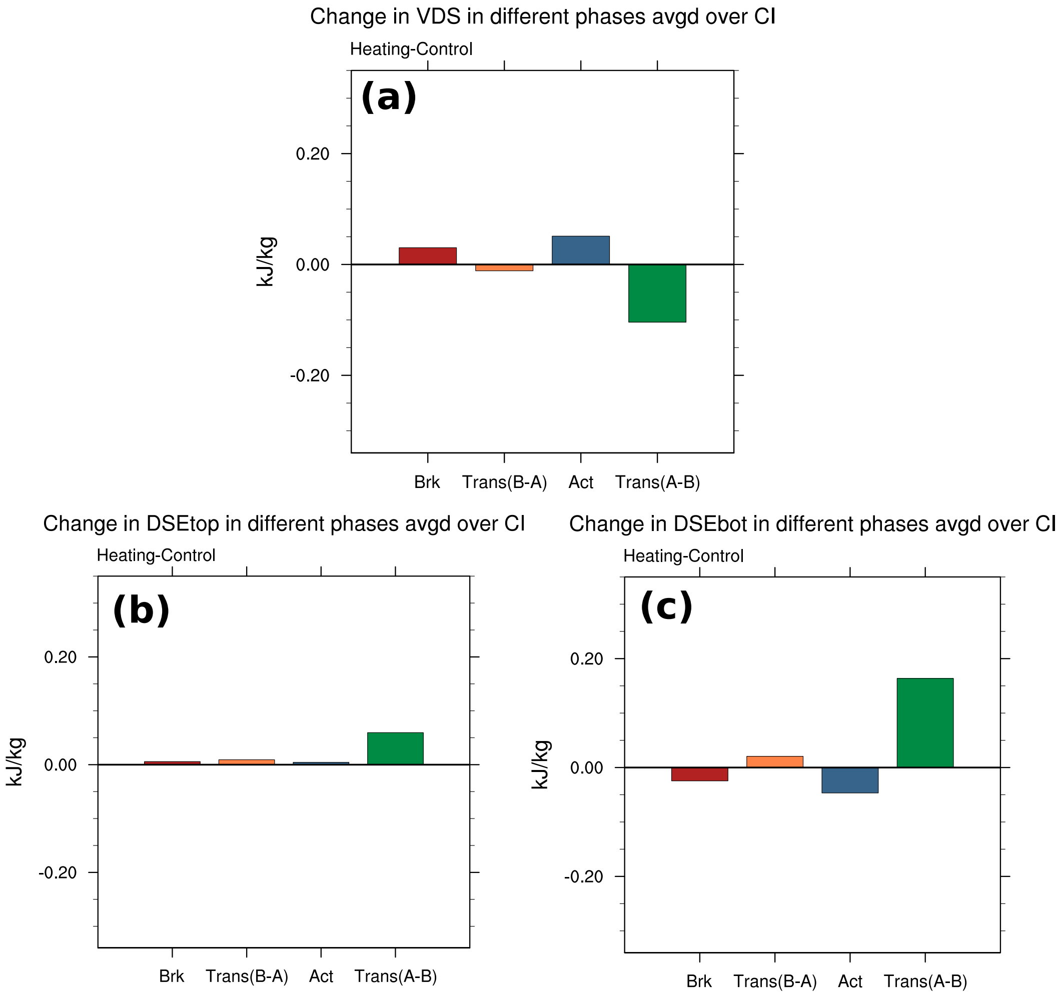

Figure S18: **Change in VDS over CI in different phases.** **(a)** Same as Fig. 3e in the main text, but for VDS. **(b)–(c)** Same as Fig. S17a and b, but for VDS. Figures are generated using NCAR Command Language 6.3.0 (http://www.ncl.ucar.edu/).

different vertical levels in active phase (Figure S19). Advection of moisture by low level monsoonal westerlies is a major source of moisture into the Indian region. It is observed that there is a significant decrease in the net moisture flux through this region in the HE, especially in the lower troposphere. This supports the fact that the change in stability of the atmosphere in the active phase of the HE is associated with the reduction of moisture supply to the Indian region during that phase.

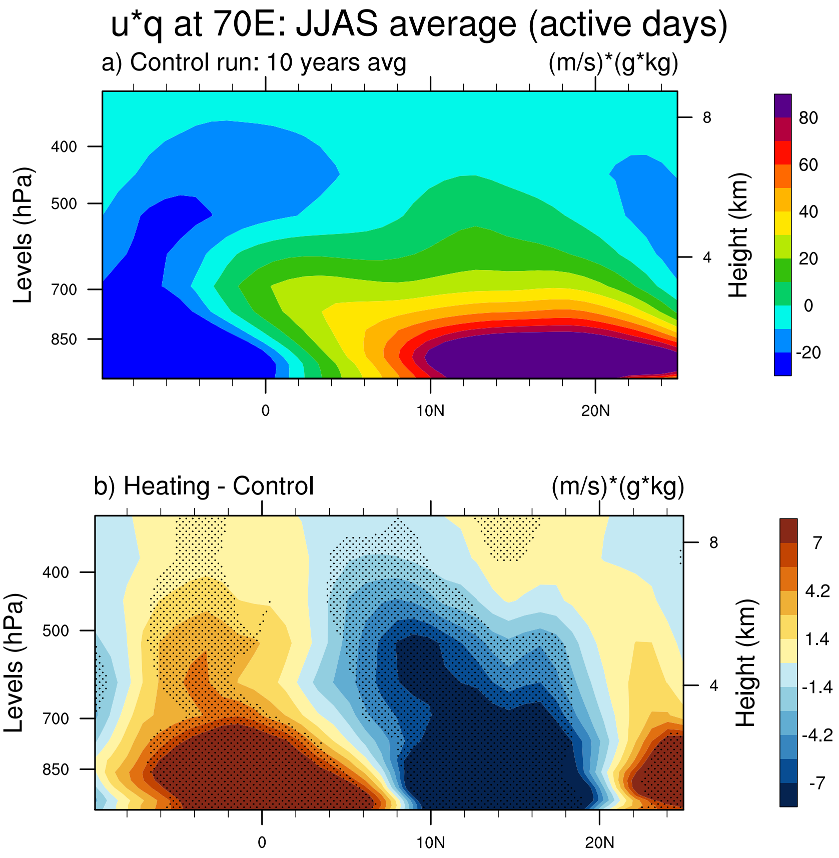

Figure S19: **Zonal moisture flux at the Arabian Sea in the control and the heating experiments.** **(a)** Moisture flux at 70°E during active phases of LF-ISO in the CE. **(b)** Change in the moisture flux in the HE compared to the CE. The stippling indicates that the differences are significant at 10% level (using paired t-test). Units are in $(m/s) * (g/kg)$. Figures are generated using NCAR Command Language 6.3.0 (`http://www.ncl.ucar.edu/`).

7 Analysis of high-frequency ISO (HF-ISO)

Similar to LF-ISO mode, we have done analysis with the HF-ISO (10–20-day) mode of rainfall and found that this mode has not shown significant changes in its variance in observation over last few decades (Fig. S20) [1]. Consistent with the observations, model also does not show significant changes in intensity in this scale after implementing heating profile to increase extreme events. However, the changes in spatial patterns differ in some places, for example, over the Western Ghats, central India (statistically not significant at 10% level).

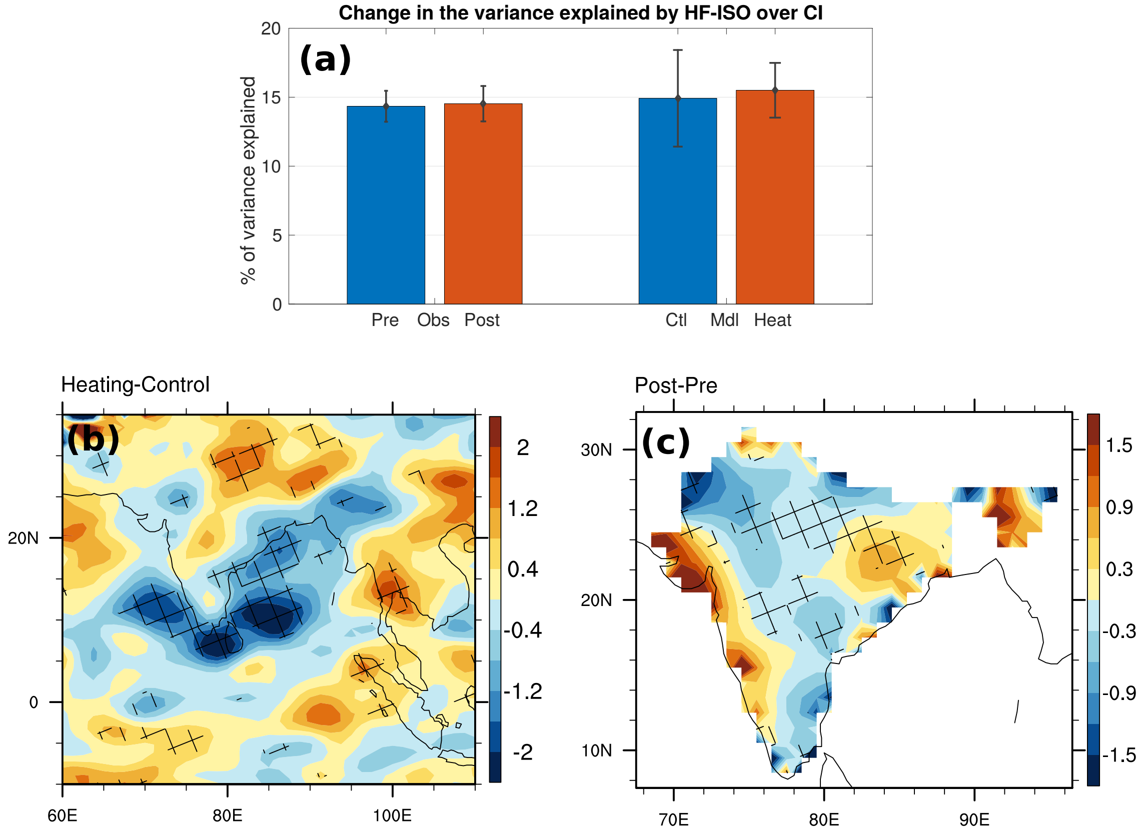

Figure S20: **Change in strength of HF-ISO mode.** Same as Fig. 2 in main text, but for HF-ISO (10–20-day mode). Figure (a) is generated using MATLAB R2015a (https://in.mathworks.com/products/new_products/release2015a.html). Figures (b) and (c) are generated using NCAR Command Language 6.3.0 (http://www.ncl.ucar.edu/).

8 Changes in global precipitation

Increase in the extremes over India shows an impact over other monsoonal regions as well (Fig. S21). For example, monsoon rainfall over Sahel and parts of east Asia has increased. Many recent observational studies indicate that Sahel monsoon has significantly increased in recent time after several consequent droughts in 1970s and 1980s [22, 23, 24]. East Asian summer monsoon also shows large variability in interannual scale and shows an increasing trend in recent time [25]. Coupled Model Intercomparison Project (CMIP5) models also show an increase in rainfall over the Baiu region and to the north and northeast of the Korean Peninsula in the future projections [26]. These results are consistent with the findings of our study, indicating a strong association between the Indian and other monsoonal systems and changes in the Indian monsoon system could affect the

global rainfall patterns. However, how these changes occur could be a topic of rigorous observational and modeling research.

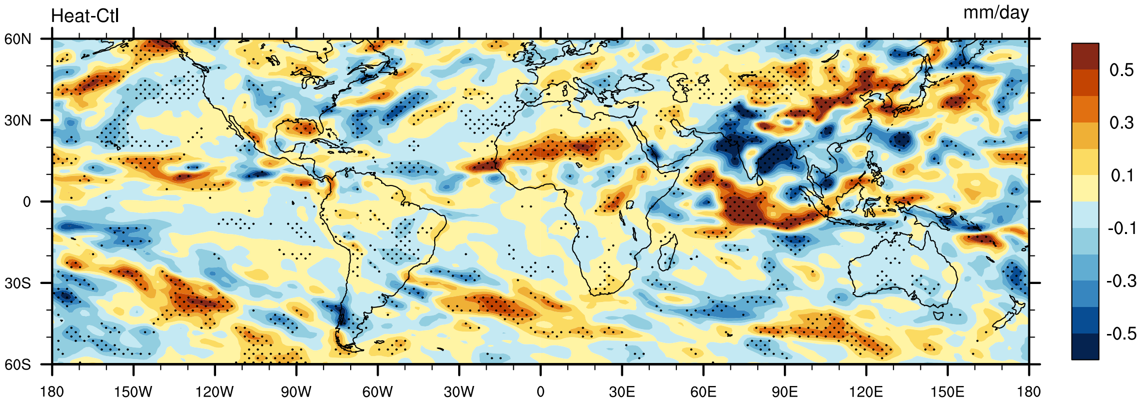

Figure S21: **Change in global precipitation.** Change (Heating − Control) in the June–September daily average rainfall in a global perspective in the model. Units are in mm/day. Figure is generated using NCAR Command Language 6.3.0 (http://www.ncl.ucar.edu/).

9 Comparison of Active/Break phases with earlier studies

There are many studies that identified active and break phases of monsoon rainfall over CI [27]. [27] suggested a criteria for the identification of active and break events of the Indian summer monsoon during only July and August months (1951–2007). Active and break events are defined as periods during "which the normalized anomaly of the rainfall over a critical area, called the monsoon core zone exceeds 1 or is less than -1.0 respectively, provided the criterion is satisfied for at least three consecutive days." By definition, the breaks and active phases are event specific and imposes a strict criteria on selecting the active/break phases. Compared to that, our active/break phases are based on the oscillatory behaviour of the LF-ISO mode. Active and breaks are phases of the LF-ISO mode averaged over a specific area (central India).

To find how our active and break phases are different than theirs, we have done an analysis on the similar time span (1951–2007) for the months of July–August. We found that the number of active and break days in their paper is lesser than that of ours, which is not surprising (Figure S22). Importantly, majority of the break days (81%) and active

days (77%) in their paper are in the intersection with the break and active days as we have defined. In spite of the fact that the region of analysis in both the studies and definition of active/break phases are somewhat different, this agreement is quite remarkable.

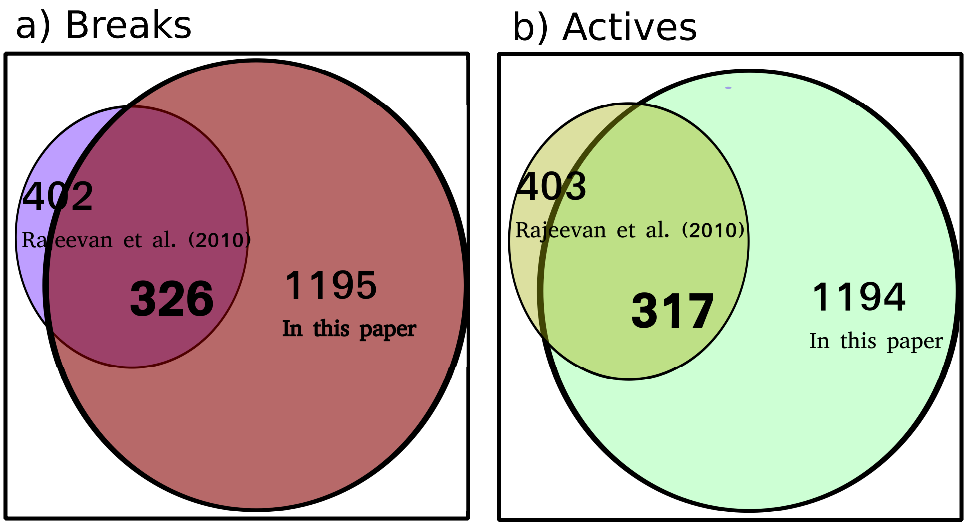

Figure S22: **Comparison of active/break phases.** **(a)** Break and **(b)** Active phases identified by [27] and in this paper in Venn-Diagrams. Numbers indicate the breaks and actives as identified in both papers. Numbers in bold and italics indicate the amount belonging to the intersections. Figure is generated using Inkscape (https://inkscape.org/en/).

References

[1] Karmakar N, Chakraborty A, Nanjundiah RS. Decreasing intensity of monsoon low-frequency intraseasonal variability over India. Environ Res Lett. 2015;10(5):054018.

[2] Hurrell JW, Holland MM, Gent PR, Ghan S, Kay JE, Kushner P, et al. The Community Earth System Model: A Framework for Collaborative Research. Bull Amer Meteor Soc. 2013;94(9):1339–1360.

[3] Lappen CL, Schumacher C. Heating in the tropical atmosphere: what level of detail is critical for accurate MJO simulations in GCMs? Climate Dyn. 2012;39(9-10):2547–2568.

[4] Lappen CL, Schumacher C. The role of tilted heating in the evolution of the MJO. J Geophys Res-Atmos. 2014;119(6):2966–2989.

[5] Jang Y, Straus DM. The Indian monsoon circulation response to El Nino diabatic heating. J Climate. 2012;25(21):7487–7508.

[6] Jang Y, Straus DM. Tropical stationary wave response to ENSO: Diabatic heating influence on the Indian summer monsoon. J Atmos Sci. 2013;70(1):193–222.

[7] Sperber KR, Annamalai H, Kang IS, Kitoh A, Moise A, Turner A, et al. The Asian summer monsoon: an intercomparison of CMIP5 vs. CMIP3 simulations of the late 20th century. Climate Dyn. 2013;41(9-10):2711–2744.

[8] Jourdain NC, Gupta AS, Taschetto AS, Ummenhofer CC, Moise AF, Ashok K. The Indo-Australian monsoon and its relationship to ENSO and IOD in reanalysis data and the CMIP3/CMIP5 simulations. Climate Dyn. 2013;41(11-12):3073–3102.

[9] Sabeerali C, Ramu Dandi A, Dhakate A, Salunke K, Mahapatra S, Rao SA. Simulation of boreal summer intraseasonal oscillations in the latest CMIP5 coupled GCMs. J Geophys Res-Atmos. 2013;118(10):4401–4420.

[10] Sooraj K, Terray P, Xavier P. Sub-seasonal behaviour of Asian summer monsoon under a changing climate: assessments using CMIP5 models. Climate Dyn. 2015;p. 1–23.

[11] Meehl GA, Arblaster JM, Collins WD. Effects of black carbon aerosols on the Indian monsoon. J Climate. 2008;21(12):2869–2882.

[12] Collier JC, Zhang GJ. Aerosol direct forcing of the summer Indian monsoon as simulated by the NCAR CAM3. Climate Dyn. 2009;32(2-3):313–332.

[13] Vinoj V, Rasch PJ, Wang H, Yoon JH, Ma PL, Landu K, et al. Short-term modulation of Indian summer monsoon rainfall by West Asian dust. Nature Geosci. 2014;7(4):308–313.

[14] Rienecker MM, Suarez MJ, Gelaro R, Todling R, Bacmeister J, Liu E, et al. MERRA: NASA's modern-era retrospective analysis for research and applications. J Climate. 2011;24(14):3624–3648.

[15] Karmakar N, Chakraborty A, Nanjundiah RS. SpaceTime Evolution of the Low- and High-Frequency Intraseasonal Modes of the Indian Summer Monsoon. Mon Wea Rev. 2017;145(2):413–435.

[16] Goswami B, Venugopal V, Sengupta D, Madhusoodanan M, Xavier PK. Increasing trend of extreme rain events over India in a warming environment. Science. 2006;314(5804):1442–1445.

[17] Neelin JD, Held IM. Modeling tropical convergence based on the moist static energy budget. Mon Wea Rev. 1987;115(1):3–12.

[18] Srinivasan J, Smith GL. Meridional migration of tropical convergence zones. J Appl Meteor. 1996;35(8):1189–1202.

[19] Chakraborty A, Nanjundiah RS, Srinivasan J. Theoretical aspects of the onset of Indian summer monsoon from perturbed orography simulations in a GCM. Ann Geophys. 2006;24(8):2075–2089.

[20] Nanjundiah RS, Srinivasan J. Anomalies of precipitable water vapour and vertical stability during El Nino. Geophys Res Lett. 1999;26(1):95–98.

[21] Chakraborty A, Nanjundiah RS, Srinivasan J. Local and remote impacts of direct aerosol forcing on Asian monsoon. Int J Climatol. 2014;34(6):2108–2121.

[22] Munemoto M, Tachibana Y. The recent trend of increasing precipitation in Sahel and the associated inter-hemispheric dipole of global SST. Int J of Climatol. 2012;32(9):1346–1353.

[23] Giannini A, Salack S, Lodoun T, Ali A, Gaye A, Ndiaye O. A unifying view of climate change in the Sahel linking intra-seasonal, interannual and longer time scales. Environ Res Lett. 2013;8(2):024010.

[24] Park Jy, Bader J, Matei D. Anthropogenic Mediterranean warming essential driver for present and future Sahel rainfall. Nature Clim Change. 2016;.

[25] Ha KJ, Heo KY, Lee SS, Yun KS, Jhun JG. Variability in the East Asian monsoon: a review. Meteorol Appl. 2012;19(2):200–215.

[26] Seo KH, Ok J, Son JH, Cha DH. Assessing future changes in the East Asian summer monsoon using CMIP5 coupled models. J of Climate. 2013;26(19):7662–7675.

[27] Rajeevan M, Gadgil S, Bhate J. Active and break spells of the Indian summer monsoon. J Earth Syst Sci. 2010;119(3):229–247.
